# Unraveling the genomic regions controlling the seed vigour index, root growth parameters and germination per cent in rice

**DOI:** 10.1371/journal.pone.0267303

**Published:** 2022-07-26

**Authors:** Saumya Ranjan Barik, Elssa Pandit, Priyadarshini Sanghamitra, Shakti Prakash Mohanty, Abhisarika Behera, Jyotirmayee Mishra, Deepak Kumar Nayak, Ramakrushna Bastia, Arpita Moharana, Auromira Sahoo, Sharat Kumar Pradhan

**Affiliations:** 1 ICAR-National Rice Research Institute, Cuttack, Odisha, India; 2 Fakir Mohan University, Balasore, India; 3 College of Agriculture, OUAT, Bhubaneswar, Odisha, India; Government College University Faisalabad, PAKISTAN

## Abstract

High seed vigour ensures good quality seed and higher productivity. Early seedling growth parameters indicate seed vigour in rice. Seed vigour *via* physiological growth parameters is a complex trait controlled by many quantitative trait loci. A panel was prepared representing a population of 274 rice landraces by including genotypes from all the phenotypic groups of sixseedling stage physiological parameters including germination % for association mapping. Wide variations for the six studiedtraits were observed in the population. The population was classified into 3 genetic groups. Fixation indices indicated the presence of linkage disequilibrium in the population. The population was classified into subpopulations and each subpopulation showed correspondence with the 6 physiological traits. A total of 5 reported QTLs *viz*., *qGP8*.*1* for germination % (GP); *qSVII2*.*1*, *qSVII6*.*1* and *qSVII6*.*2* for seed vigour index II (SVII), and *qRSR11*.*1* for root-shoot ratio (RSR) were validated in this mapping population. In addition, 13 QTLs regulating the physiological parameters such as *qSVI 11*.*1* for seed vigour index I; *qSVI11*.*1* and *qSVI12*.*1* for seed vigour index *II; qRRG10*.*1*, *qRRG8*.*1*, *qRRG8*.*2*, *qRRG6*.*1* and *qRRG4*.*1* for rate of root growth (RRG); *qRSR2*.*1*, *qRSR3*.*1* and *qRSR5*.*1* for root-shoot ratio (RSR) while *qGP6*.*2* and *qGP6*.*3* for germination %were identified. Additionally, co-localization or co-inheritance of QTLs, *qGP8*.*1 and qSVI8*.*1* for GP and SVI-1; *qGP6*.*2* and *qRRG6*.*1* for GP and RRG, and *qSVI11*.*1* and *qRSR11*.*1 for SVI and* RSR were detected. The QTLs identified in this study will be useful for improvement of seed vigour trait in rice.

## Introduction

Rice is the most important cereal crop for human consumption as more than half the global population uses this as staple food and hence there is a need to increase the production [1]. Food requirement for the increasing human population, theglobal demand is expected to reach almost double by 2050 which is a big challenge under the adverse effects of the climate change [2,3]. A good quality seed not only ensures high yield but also considered as a mega factor for enhancing crop productivity [4]. The genetic potential of a plant variety is fully manifested by use of quality seed which is considered as the basic and most vital input for crop production. Seed vigour is one of the most important determinant of seed quality, directly influences the crop productivity by delivering the genetic and yield potentials of the seed while ensuring uniformity in seed germination, seedling growth, establishment of seedling in the field and withstanding unfavourable environmental condition [5,6]. In addition, high seed vigour is also equally important for direct seeding as it enhances early crop establishment [7,8] and produces vigorous seedling that can compete with weeds [9,10]. Improving the seed vigour of rice remains a breeding challenge [11] as it is not only essential to enhance the yield but also can improve crop resilience against adverse effects of climate change and biotic impediments to crop yields [12].

Seed vigour *via* physiological growth parameters is a complex trait controlled by many quantitative trait loci (QTLs). To investigate the inheritance of such complex trait, the QTL analysis has successfully proven as a powerful tool in the last decades. The detected QTLs for rice seed vigour were associated with many physiological traits *viz*., root length, shoot length, dry weight of seedling, germination rate, radicle length, root activity, coleoptile length, mesocotyl length, germination potential, germination index and time for 50% germination [13–25]. The QTLs reported for seed vigour in rice were associated with many physiological traits *viz*., root length, shoot length, dry weight of seedling, germination rate, radicle length, root activity, coleoptile length, mesocotyl length, germination potential, germination index and time for 50% germination [13–25]. Many QTLs controlling germination % in rice were reported by earlier researchers [17–19,26–32]. Good seedling root features playimportant role in water and nutrient absorption from the soil and support to the plant. However, information on the vigour related seedling root mapping studies are limited [15,17,23,33–35]. The rapid growth of seedlings after germination is an important feature of good seedling vigour. Also a limited number of mapping information on shoot length and its related parameters are available [25–38]. However, the QTLs reported for the physiological traits described above are not always consistent for the mapped position.

Majority of the genes/QTLs reported for seed vigour in rice were based on bi-parental segregating populations. Association mapping based on linkage disequilibrium (LD) is a very successful approach of complex traits mapping. In addition, the QTL identifiedin this approachshowbetter resolution by utilizing the available natural variation [3,39,40]. The microsatellite markers are widely used to assess genetic diversity and genetic structure in rice as they are hyper variable, multi-allelic in nature,robust, co-dominant, chromosome specific and greatly facilitate linkage map construction [41–45]. Population genetic structure is useful in detecting a perfect marker-phenotype association. The population structure (Q) with relative kinship (K) analyses is used to check and correct the panel population composition for linkage disequilibrium (LD) mapping analyses [46,47]. Association analysis based on both the models of General linear model (GLM) and Mixed linear model (MLM)is considered appropriate for mapping complex traits that have shown to perform better than other models analysis. However, very limited information is available on genetic analysis of seedling stage physiological parameters related to seed vigour in a variable rice natural population.

In the present investigation, association mapping of seedling growth parameters including germination %with SSR markers was performed in a representative shortlisted population by including genotypes from all the phenotypic classesfor the early seedling growth parameters namely seed vigour index I, seed vigour index II, relative root growth, root shoot ratio, rate of plumule elongationand germination per cent from a total of 274rice landraces. The study will reveal the population genetic structure, diversity and candidate genes/QTLs controlling the 6seedling growth parameter associated with seed vigour in rice.

## Material and methods

### Seed material

The freshly harvested seeds of 274 landraces collected from five states *viz*., Assam, MP, Kerala, Odisha and Manipur of India were used for association mapping of seedling stage physiological parameters with molecular markers. The germplasm lines of Odisha state were from the Jeypore tract, the secondary center of origin of rice and known for availability of rich diversity of rice. All the germplasm lines were collected from Gene bank, ICAR-NRRI, Cuttack and grown during wet season, 2018 (S1 Table). The harvested seeds were used for estimation of 6seedling growth parameters after a storage period of three months to overcome the seed dormancy. A panel population was developed and raised during the wet seasons of 2019 and 2020. Thepanel population comprising 120landraceswas used for mapping of the 6growth parameters including germination % (Table 1).

**Table 1:** Mean estimates of SVI-1, SVI-II, RRG, RPE, RSR, GP and genetic structure ancestry value at K = 3 for the panel population containing 120 landraces.

Sl.No.	Accession No./Name of the germplasm line	SVI-I	SVI-II	RRG	RPE	RSR	GP	Inferred ancestry value at K = 3			Genetic structure group
								Q1	Q2	Q3	
1	Jhagrikartik	202.12	0.057	0.970	0.53	2.148	20.00	0.008	0.99	0.002	SP2
2	Dadghani	357.40	0.137	0.110	0.15	0.933	50.00	0.005	0.992	0.003	SP2
3	Shayam	676.52	0.127	0.810	0.24	1.460	78.00	0.001	0.002	0.996	SP3
4	Basumati	481.60	0.100	0.290	0.29	0.888	80.00	0.005	0.113	0.883	SP3
5	Bharati	131.64	0.070	0.127	0.19	0.778	14.00	0.001	0.997	0.002	SP2
6	Joha	103.60	0.100	0.220	0.20	1.151	20.00	0.002	0.997	0.002	SP2
7	Adira-1	380.12	0.216	0.230	0.40	0.962	28.00	0.019	0.619	0.362	A
8	Adira-2	1273.20	0.592	0.753	0.43	1.074	84.00	0.002	0.997	0.001	SP2
9	Adira-3	602.68	0.293	0.533	0.21	1.133	50.00	0.003	0.593	0.403	A
10	PK6	400.24	0.178	0.463	0.15	1.782	46.00	0.004	0.982	0.014	SP2
11	Vachaw	523.72	0.316	0.463	0.19	1.390	74.00	0.002	0.961	0.037	SP2
12	Kozhivalan	571.12	0.355	0.093	0.31	1.076	66.00	0.002	0.997	0.001	SP2
13	Marathondi	264.20	0.112	0.360	0.37	0.652	26.00	0.034	0.516	0.45	A
14	Ezhoml-2	526.84	0.215	0.243	0.05	1.079	74.00	0.001	0.998	0.001	SP2
15	Jyothi	190.40	0.112	0.423	0.19	1.088	28.00	0.001	0.998	0.001	SP2
16	Kantakopura	885.20	0.525	0.447	0.72	0.891	70.00	0.001	0.997	0.002	SP2
17	Kantakaamala	713.20	0.419	0.640	0.67	0.987	68.00	0.109	0.675	0.216	A
18	Kapanthi	1349.76	0.684	0.677	0.54	1.366	76.00	0.341	0.304	0.355	A
19	Karpurkanti	661.12	0.151	0.403	0.55	0.842	70.00	0.001	0.001	0.998	SP3
20	Kathidhan	523.20	0.327	0.420	0.63	1.118	38.00	0.097	0.898	0.005	SP2
21	Kundadhan	213.92	0.076	0.157	0.38	0.907	20.00	0.005	0.994	0.001	SP2
22	Champaeisiali	1011.72	0.479	1.113	0.80	1.403	58.00	0.003	0.993	0.004	SP2
23	Latamahu	318.48	0.182	0.830	0.81	1.074	28.00	0.003	0.996	0.002	SP2
24	Latachaunri	633.20	0.303	0.417	0.50	1.336	64.00	0.005	0.993	0.002	SP2
25	AC5993 (Mikirahu)	88.16	0.248	0.260	0.35	1.320	16.00	0.003	0.995	0.003	SP2
26	AC6221(Ana pachidhan)	483.56	0.310	0.617	0.16	1.309	70.00	0.007	0.991	0.002	SP2
27	AC6183 (Kanai muluk)	451.44	0.198	1.617	0.31	2.185	36.00	0.072	0.925	0.003	SP2
28	AC6170 (Bengali joha)	532.16	0.179	0.447	0.20	1.170	84.00	0.003	0.995	0.002	SP2
29	AC6023 (Tili bora)	151.84	0.160	1.070	0.38	1.380	16.00	0.02	0.978	0.002	SP2
30	AC6172 (ManoharSali)	684.80	0.490	0.600	0.21	1.184	80.00	0.051	0.948	0.002	SP2
31	AC6027 (Chingforechokua)	141.92	0.134	0.553	0.27	1.004	28.00	0.002	0.005	0.993	SP3
32	AC6007 (Manipur local)	323.28	0.093	0.973	0.21	1.203	30.00	0.004	0.995	0.001	SP2
33	AC9006 (Aujari)	717.36	0.583	0.736	0.16	0.896	45.00	0.02	0.971	0.009	SP2
34	AC9021 (Kabokphou)	865.33	0.477	0.240	0.23	0.907	58.33	0.013	0.982	0.005	SP2
35	AC9028 (Mayangkhang-I)	459.00	0.637	0.923	0.06	0.814	30.00	0.069	0.928	0.003	SP2
36	AC9030 (Moiranghouanganba)	514.27	0.898	0.527	0.10	0.596	35.00	0.006	0.994	0.001	SP2
37	AC9035 (Taothali)	397.80	0.515	0.917	0.14	0.585	25.00	0.004	0.983	0.014	SP2
38	AC9038 (Mayangkhang-II)	891.87	1.020	0.903	0.20	0.804	53.33	0.001	0.998	0.001	SP2
39	AC9043 (Phoudum)	799.87	1.123	0.280	0.13	0.744	50.00	0.002	0.997	0.001	SP2
40	AC9044 (Changli)	690.00	0.740	1.247	0.09	0.919	40.00	0.003	0.993	0.005	SP2
41	AC20920 (Pondremunduria)	771.67	0.504	1.133	0.26	0.884	60.00	0.003	0.993	0.004	SP2
42	AC20907 (Lalmunduria)	318.60	0.168	0.267	0.41	0.854	30.00	0.002	0.997	0.001	SP2
43	AC20845 (Jhitikuji)	629.84	0.453	0.510	0.14	0.829	42.00	0.003	0.996	0.001	SP2
44	AC20770 (Tikichudi)	842.24	0.336	0.287	0.25	0.725	56.00	0.002	0.993	0.005	SP2
45	AC20627 (Chudi)	492.52	0.212	0.227	0.37	0.696	42.00	0.002	0.997	0.001	SP2
46	AC20686 (Radhabati)	169.16	0.048	0.147	0.20	0.863	16.00	0.002	0.997	0.001	SP2
47	AC20664 (Liktimachi)	121.84	0.078	0.220	0.43	0.821	16.00	0.003	0.996	0.001	SP2
48	AC20614(Baranga)	371.20	0.245	0.320	0.41	1.066	40.00	0.001	0.996	0.002	SP2
49	AC10608 (Ampang)	320.56	0.175	0.440	0.12	1.093	38.00	0.006	0.994	0.001	SP2
50	AC10187 (Mimahambel)	1060.20	0.412	1.917	0.22	1.598	80.00	0.081	0.917	0.002	SP2
51	AC10162 (Ahimachutki)	256.24	0.071	1.067	0.35	1.272	28.00	0.023	0.96	0.017	SP2
52	AC7282 (Mimagisim)	166.32	0.074	1.137	0.35	1.025	28.00	0.001	0.002	0.997	SP3
53	AC7269 (Champalidhan)	113.00	0.194	0.570	0.66	1.124	18.00	0.002	0.997	0.001	SP2
54	AC7134 (Memabalbok)	142.72	0.140	0.687	0.29	1.186	20.00	0.009	0.793	0.198	A
55	AC7008 (Kartiksal)	129.76	0.088	0.660	0.11	1.071	16.00	0.002	0.998	0.001	SP2
56	AC9093 (Turnaianganba)	446.16	1.298	0.457	0.37	0.811	44.00	0.001	0.995	0.004	SP2
57	AC9090 (Chakhaosimpak)	451.36	1.050	0.477	0.42	1.354	38.00	0.004	0.987	0.01	SP2
58	AC9076A(Phoaujaarangbele)	173.76	0.180	0.543	0.58	1.669	12.00	0.006	0.993	0.001	SP2
59	AC9065 (Moirangphon)	498.24	1.094	0.270	0.38	1.054	46.00	0.003	0.963	0.034	SP2
60	AC9063 (Chingphou)	326.12	0.856	0.367	0.11	0.917	34.00	0.007	0.992	0.001	SP2
61	AC9058 (Langmanbu)	332.96	0.962	0.237	0.39	0.816	38.00	0.001	0.998	0.001	SP2
62	AC9053A(Kakchengphou)	105.28	0.080	0.140	0.27	0.756	16.00	0.153	0.839	0.008	SP2
63	AC9050 (Phongangangamphou)	261.08	0.564	0.217	0.25	1.027	34.00	0.002	0.994	0.004	SP2
64	AC9005 (Phourrel)	849.97	0.563	0.803	0.29	1.172	48.33	0.004	0.993	0.002	SP2
65	AC20389 (Kusumal)	999.32	0.908	0.513	0.27	0.809	80.00	0.046	0.943	0.011	SP2
66	AC20371 (Kakudimanji)	1356.84	1.318	0.347	0.19	0.763	94.00	0.007	0.992	0.001	SP2
67	AC20423 (Salati)	475.64	0.204	1.090	0.21	1.071	32.00	0.003	0.996	0.001	SP2
68	AC20362 (Kaberi)	1129.04	0.886	0.377	0.55	0.818	84.00	0.018	0.975	0.007	SP2
69	AC20328 (Batachudi)	1255.68	1.161	0.540	0.34	0.796	86.00	0.009	0.979	0.012	SP2
70	AC20317 (Barda)	569.16	0.442	0.347	0.33	0.999	68.00	0.003	0.973	0.024	SP2
71	AC20282 (Mahipaljeera)	1149.28	0.789	0.883	0.42	0.805	70.00	0.108	0.886	0.006	SP2
72	AC20246 (BodiKaberi)	660.96	0.452	0.403	0.58	0.685	48.00	0.024	0.89	0.086	SP2
73	AC20347 (Dudhamani)	310.60	0.138	0.563	0.38	0.809	28.00	0.066	0.932	0.001	SP2
74	AC44603 (Sonamasuri)	1490.00	1.120	1.250	0.50	1.678	100.00	0.985	0.014	0.001	SP1
75	AC44585 (Bilijaya)	1396.67	2.190	1.083	0.80	0.906	100.00	0.989	0.003	0.008	SP1
76	AC44598 (Jira)	835.11	0.608	1.300	0.45	1.332	66.67	0.988	0.007	0.006	SP1
77	AC44592 (Malbar)	1900.00	1.630	1.133	0.40	1.286	100.00	0.991	0.001	0.008	SP1
78	AC44646 (Jaya Padma)	1621.44	1.709	1.567	0.70	1.164	96.67	0.998	0.001	0.001	SP1
79	AC44604 (Lusai)	1526.67	2.720	0.150	0.40	1.399	100.00	0.977	0.013	0.01	SP1
80	AC44597 (Bilipandya)	1890.33	2.804	1.200	0.35	0.985	96.67	0.997	0.002	0.001	SP1
81	AC44638 (Kalame)	1536.00	0.875	2.583	0.50	1.231	93.33	0.29	0.001	0.71	A
82	AC44595 (Chitapa)	1664.44	1.699	0.283	0.70	0.711	93.33	0.99	0.004	0.006	SP1
83	AC44588 (Badra)	1420.00	1.940	0.250	0.50	0.841	100.00	0.997	0.002	0.001	SP1
84	AC44591 (Gouri)	1443.33	1.050	0.333	0.50	0.904	100.00	0.998	0.002	0.001	SP1
85	AC44594 (Pandya)	1986.67	2.430	1.350	0.70	0.789	100.00	0.99	0.008	0.002	SP1
86	AC43737 (karinellu)	1498.33	0.920	3.833	0.60	0.854	70.00	0.996	0.002	0.002	SP1
87	AC43660 (Manavari)	1215.50	0.625	3.422	0.40	0.727	45.00	0.997	0.002	0.001	SP1
88	AC3732 (Koompallai)	1163.50	1.050	2.550	0.20	0.838	80.00	0.998	0.001	0.001	SP1
89	AC43661 (MDU-5)	737.83	0.443	2.583	0.10	0.925	45.00	0.995	0.004	0.001	SP1
90	AC43738 (Belimuruduga)	668.33	0.740	2.200	0.40	1.165	50.00	0.996	0.002	0.002	SP1
91	AC43669 (Tulasi)	1809.67	0.950	3.533	0.35	0.982	70.00	0.994	0.004	0.002	SP1
92	AC43663 (TKM10)	1041.33	0.890	1.200	0.40	0.587	70.00	0.997	0.001	0.002	SP1
93	AC43658 (Noorthipathu)	758.00	0.590	3.000	0.80	0.584	50.00	0.998	0.001	0.001	SP1
94	AC43662 (PMK2)	1287.83	0.985	2.633	0.70	0.754	80.00	0.979	0.002	0.018	SP1
95	AC43670 (Aditya)	1117.41	0.567	2.583	0.65	1.130	55.56	0.827	0.002	0.171	SP1
96	AC43675 (Kalimekri 77–5)	653.33	0.480	2.383	0.20	0.759	50.00	0.99	0.002	0.007	SP1
97	AC43676 (Pratao)	1131.17	0.945	2.183	0.20	0.567	65.00	0.959	0.008	0.033	SP1
98	Palinadhan-1	102.88	0.038	0.267	0.28	1.049	16.00	0.237	0.393	0.371	A
99	Chatuimuchi	608.80	0.329	0.663	0.22	1.167	84.00	0.001	0.001	0.998	SP3
100	Uttarbangalocal-9	56.92	0.048	0.200	0.14	1.432	10.00	0.094	0.904	0.001	SP2
11	Gochi	171.96	0.120	0.250	0.09	0.884	26.00	0.065	0.927	0.008	SP2
12	Sugandha-2	540.80	0.208	0.457	0.24	1.399	84.00	0.001	0.002	0.997	SP3
13	Jhingesal	370.44	0.169	0.610	0.37	1.128	34.00	0.002	0.997	0.001	SP2
104	Cheruvirippu	411.04	0.149	0.300	0.28	1.126	56.00	0.004	0.994	0.001	SP2
105	Mahamaga	250.08	0.066	0.443	0.19	0.974	42.00	0.047	0.951	0.002	SP2
106	Jaya	442.00	0.300	0.527	0.24	1.303	64.00	0.009	0.99	0.001	SP2
107	D1	185.36	0.130	0.357	0.20	1.024	34.00	0.049	0.94	0.011	SP2
108	PK21	1151.00	0.400	0.787	0.27	0.835	100.00	0.016	0.983	0.001	SP2
109	Gandhakasala	397.76	0.152	0.720	0.14	1.387	58.00	0.003	0.004	0.993	SP3
110	Sreyas	235.36	0.127	0.667	0.19	1.158	32.00	0.002	0.995	0.002	SP2
111	Gondiachampeisiali	1010.48	0.647	0.513	0.68	0.854	66.00	0.003	0.995	0.002	SP2
112	Chinamal	637.40	0.411	0.583	0.41	1.248	46.00	0.002	0.983	0.015	SP2
113	Magra	390.36	0.138	1.213	0.09	1.392	30.00	0.002	0.995	0.003	SP2
114	Landi	672.60	0.184	0.747	0.65	1.168	46.00	0.002	0.997	0.002	SP2
115	Lalgundi	334.88	0.054	0.243	0.68	0.802	36.00	0.004	0.989	0.006	SP2
116	Balisaralaktimachi	579.96	0.244	1.193	0.51	1.264	40.00	0.003	0.993	0.003	SP2
117	Laxmibilash	257.00	0.096	0.273	0.60	0.970	22.00	0.003	0.427	0.57	A
118	Kaniar	918.28	0.619	0.407	0.56	0.799	66.00	0.005	0.981	0.015	SP2
119	Kanakchampa	277.68	0.204	0.733	0.33	0.942	24.00	0.004	0.982	0.015	SP2
120	Magura-s	400.40	0.136	0.647	0.45	0.766	32.00	0.002	0.914	0.084	SP2
	**Mean**	672.85	0.54	0.85	0.36	1.04	52.20				
	**CV%**	12.35	13.41	14.25	10.23	11.62	9.31				
	**LSD**_**5%**_	127.422	0.131	0.728	0.137	0.286	9.650				

### Phenotyping forsix seedling stage physiological traits

Seed physiological characteristics such as seed vigour index I, seed vigour index II, rate of root growth, rate of plumule elongation, root shoot ratio andgermination per cent were estimated for the mapping study. Fifty seeds were germinated in three replicationsby adopting the top of paper method of Rao et al. [48] for panel development. Plastic trays were used for germination and raising the seedlings for phenotyping of the panel population. For estimating the sixgrowth parameters, observations observation on five seedlings were recorded from each replication and averaged to get the value of each replication. The germination % (GP) is the percentage of germinated seeds at 10^th^ day was referred as the final germination percentage. Root length (RL) was measured on 10^th^ day of germination and expressed in cm. The increase in plumule length per day was considered as rate of plumule elongation (RPE) and expressed in cm day^-1^. The increase in root length per day i.e rate of root grow (RRG) recorded on 7^th^ day and 10^th^ day of germination were considered and expressed in cm day^-1^. The seedlings used for recording root:shoot ratio (RSR) were oven dried at 70 °C for 48 hours after removing the cotyledon and seedling dry weight was expressed in gram per seedling as per the protocol of Kleyer et al. [49]. Seed vigour indices (SVI I and SVI II) were calculated using the formula suggested by Abdul-Baki and Anderson [50].

Analysis of variance (ANOVA) of each traits including the estimation of mean, range, and coefficient of variation (CV %) were estimated by using Cropstat software 7.0 [51]. Pearson’s correlation coefficients were analyzed to find out the relationship among the various physiological traits, based on the mean values of the 120 genotypes and presented in correlation matrix heatmap. The mean estimates of the 6 physiological parameters were classified into 4 groups as very high, high, medium and low value containing germplasm lines for this study.

### Genomic DNA isolation, PCR analysis and selection of SSR markers

Genomic DNA of the germplasm lines was extracted from 15 days-old plant by adopting CTAB method [52]. The 136 SSR (simple sequence repeat) markers were taken from the data base available in the public domain (S1 Table). The isolated DNA was quantified by resolving the DNA fragments in gel electrophoresis. PCR analysis was done using the markers selected based on position covering all the chromosomes to illustrate the diversity and to identify the polymorphic loci among the 120 rice landraces (Table 1). Conditions of PCR reaction was set to initial denaturation step (2 min, 95°C), followed by 35 cycles of denaturation (30 s, 95°C) and annealing/extension (30 min, 55°C), extension (2 min, 72ºC), final extension (5 min, 72ºC) and store at 4ºC (infinity). The PCR products were electrophoresed using 2.5% agarose gel containing 0.80g ml^-1^ ethidium bromide. To determine the size of amplicons, 50 bp DNA ladder was used. The gel was run at 2.5V cm^-1^ for 4 hrs and photographed using a Gel Documentation System [53]. The procedures followed in earlier publications were adopted in this work [54–56].

### Molecular data analysis

Data scoring was carried out from the presence or absence of amplified products obtained on the basis genotype-primer combination. A binary data matrix was used as discrete variables for the entry of our result data. Software, ‘Power Marker Ver3.25’ was used to analyze the parameters namely polymorphic information content (PIC), observed heterozygosity (H), number of alleles (N), major allele frequency (A) and gene diversity (GD) for each SSR locus [57]. A Bayesian model based clustering approach STRUCTURE 2.3.6 software was used to analysis genetic data and obtain population structure [58]. To derive the ideal number of groups (K), STRUCTURE software was run with K varying from 1 to 10, with 10 iterations for each K value. A high throughput parameter set of burn-in period of the 150,000 followed by 150,000 Markov Chain Monte Carlo (MCMC) replications was adapted during the running period. Highest value of ΔK was pick up from Evanno table used to detect the subpopulation groups from the panel of populations in the next step. The maximal value of L(K) was identified using the exact number of sub-populations. The model choice criterion to detect the most probable value of K was ΔK, an *ad-hoc* quantity related to the second-order change of the log probability of data with respect to the number of clusters inferred by STRUCTURE. Structure Harvester was used for estimation of the ΔK-value as function of K showing a clear peak as the optimal K-value [59]. The principal coordinate analysis of all the genotypes and unweighted neighbor joining unrooted tree for NEI coefficient dissimilarity index with bootstrap value of 1,000 were obtained by using DARwin5 software [60]. The presence of molecular variance across the whole population, within a population and between the sub-population structures (F_IT_, F_IS_, F_ST_) was calculated by the deviation from Hardy-Weinberg expectation and estimated through Analysis of molecular variance (AMOVA) using GenAlEx 6.5 software [61]. All the detailed protocols of the above mentioned softwares were described in earlier publications [39,62].

Software, “TASSEL 5.0” was used to analyze the marker-trait association for mapping study of the seed vigor traits in rice. General linear model and Mixed linear model in TASSEL 5.0 were used to perform the genetic association between the phenotypic traits and molecular markers [63]. By considering the significant *p-value* and r^2^ value convincing associated markers were identified. The associations of markers were further confirmed by the Q-Q plot generated by the software. Linkage disequilibrium plot was obtained using LD measured r^2^, between pair of markers is plotted against the distance between the pair. Also, the accuracy of the marker-trait association by estimating the FDR adjusted p-values (q-values) using R software as described in the earlier publications [39,47].

## Results

### Phenotyping of the population for seedling stage physiological traits in rice

**Fig 1:**
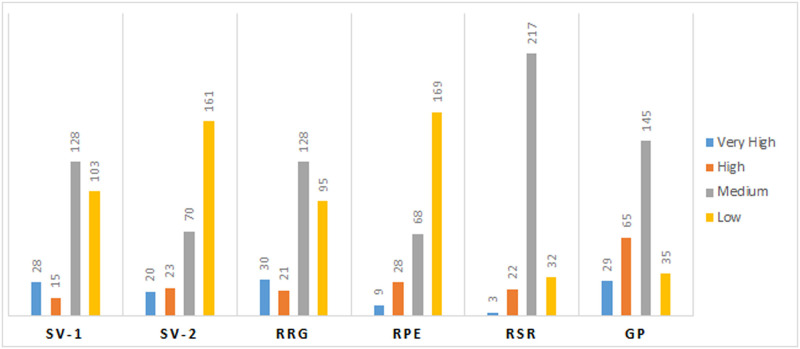
Frequency distribution of germplasm lines for each of the seedling stage physiological parameters for SVI-1, SVI-2, RRG, RPE, RSR and GP estimated from 274rice landraces.

**Fig 2:**
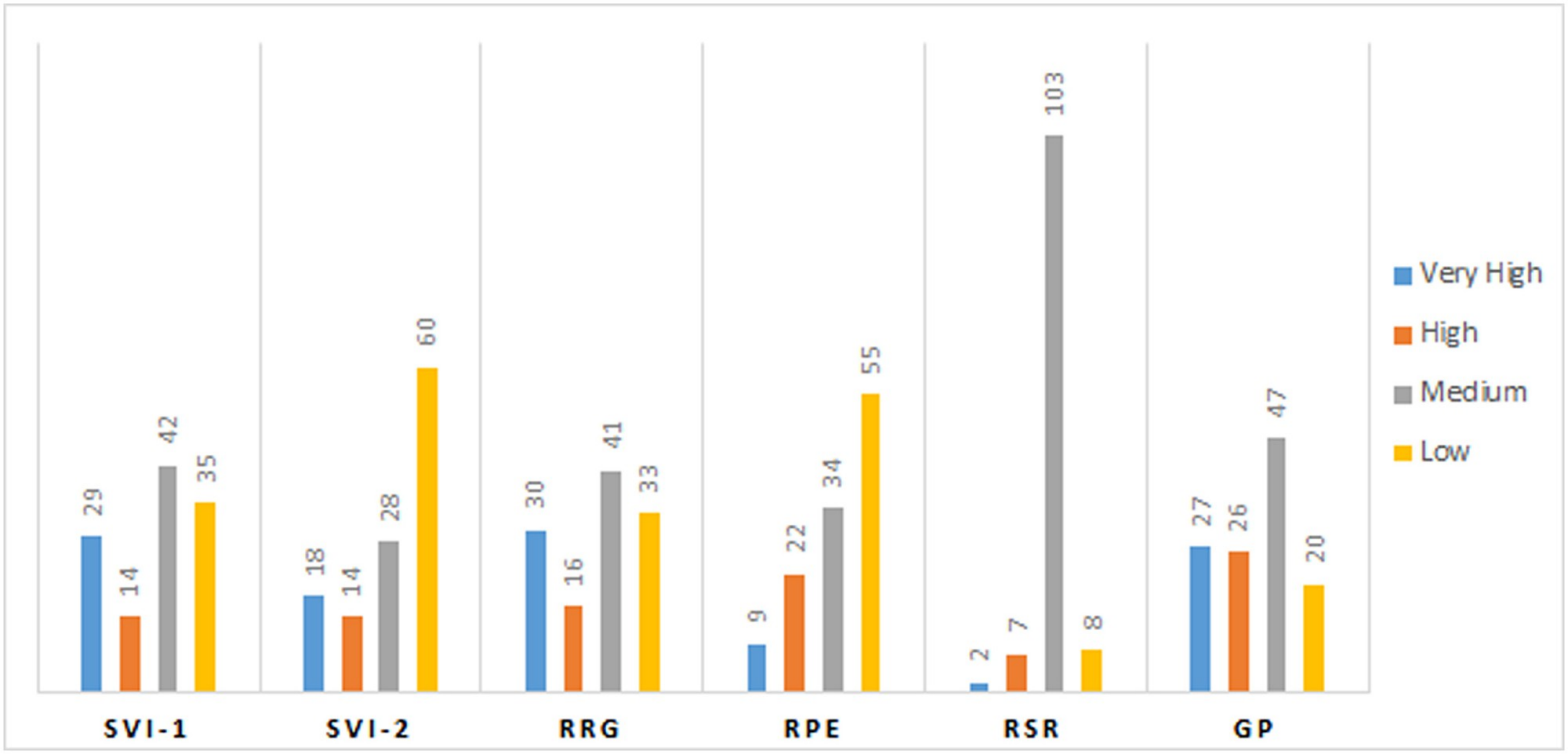
Frequency distribution of germplasm lines for each of the seedling stage physiological parameters for SVI-1, SVI-2, RRG, RPE, RSR and GP estimated from the panel population containing 120rice landraces.

**Fig 3:**
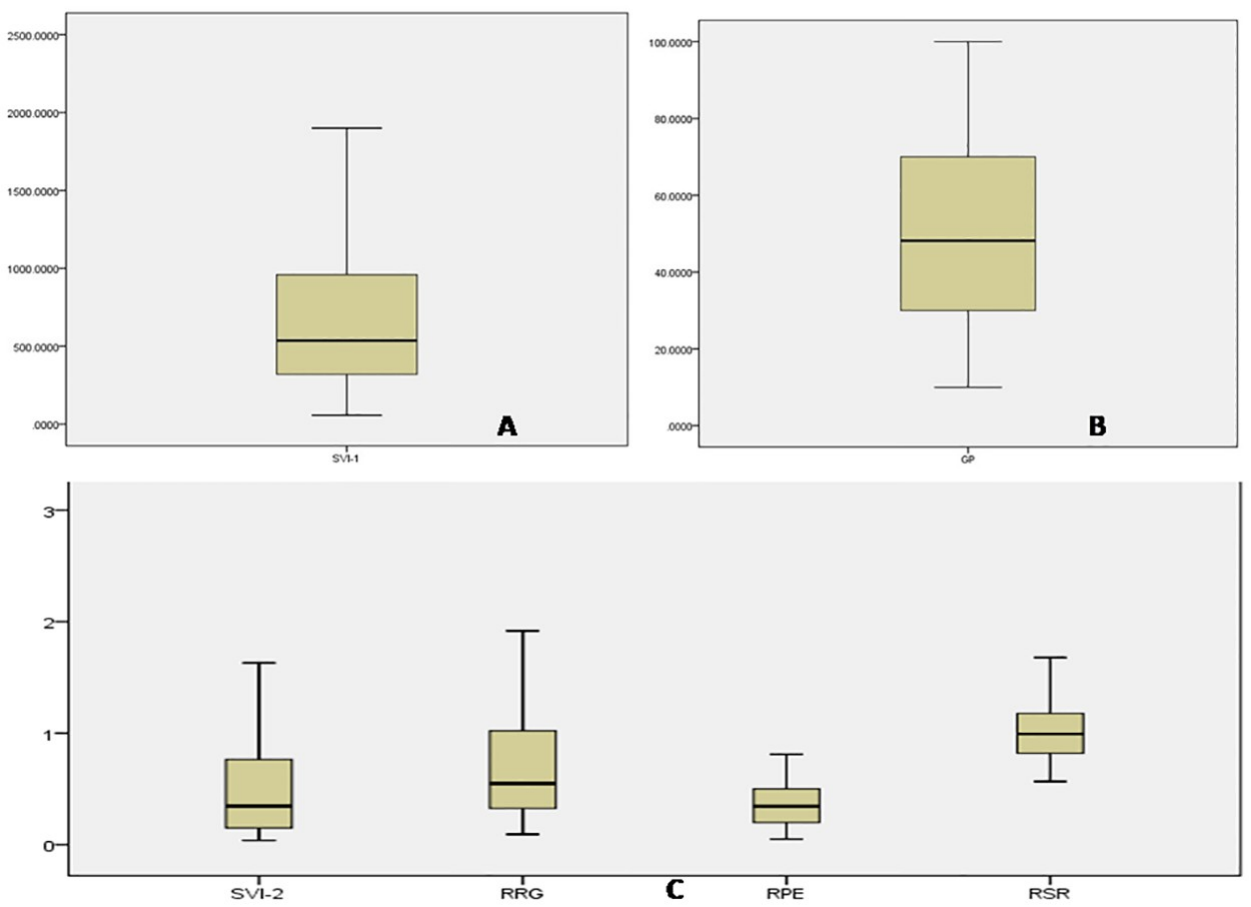
Box plots representing the phenotypic variation in the physiological parameters for (A) Seed vigour index-I (B) Germination %, (C) for Seed vigour index-II, RRG, RPE and RSR in a panel containing 120 rice landraces.

The mean values of 6 physiological traits *viz*., GP, SVI, SVII, RRG, RPE, and RGR related to seed vigour were estimated from274 landraces during the wet seasons of 2019 and 2020 (S1 Table). Significant differences were noticed among the germplasm lines for these 6 traits. The frequency distribution of the 274 germplasm lines were broadly classified into 4 groups each for the 6 physiological parameters (Fig 1). The distribution of germplasm lines into various groups were categorized into groups or subpopulations. A representative panel population containing 120 landraces was developed from the original population by shortlisting germplasm lines from all the phenotypic groups of each parameter (Table 1; Figs 2 and 3). The mean values of the 6 physiological traits estimated from the studied panel population also showed significant variation among the genotypes for each trait (Table 1). AC. 9038, AC. 9043, AC. 20282, AC. 20328, AC. 20362, AC. 20371, and AC. 20389 showed very high values for both seed vigour index I and seed vigour index II. High seed vigour index-II and germination per cent exhibited by the landraces Kapanthi, PK21, AC. 10187, AC. 20389, AC. 20371, AC. 20362 and AC. 20328. Rate of root growth and root shoot rate were high in the germplasm lines Champaisali, Jhagirikartik, Latamahu, Adira-2, AC. 6183, AC. 6023, AC. 6007, AC. 10187, AC. 10162, AC. 7282, AC. 9005 and AC. 20423. Landraces Champaisali, Jhagirikartik and Latamahu showed high value for the traits, RPE, RSR and RRG. Landraces showinghigh values for > 4 parameters identifiedfrom the landraces were Champeisali, AC. 10187, AC. 3663, AC. 44638, AC. 44604, AC. 44646, AC. 44598, AC. 20362, AC. 9038, Kapanthi and Adira-2 (Table 1).

#### Genotype-by-trait biplot and correlation analyses

**Fig 4:**
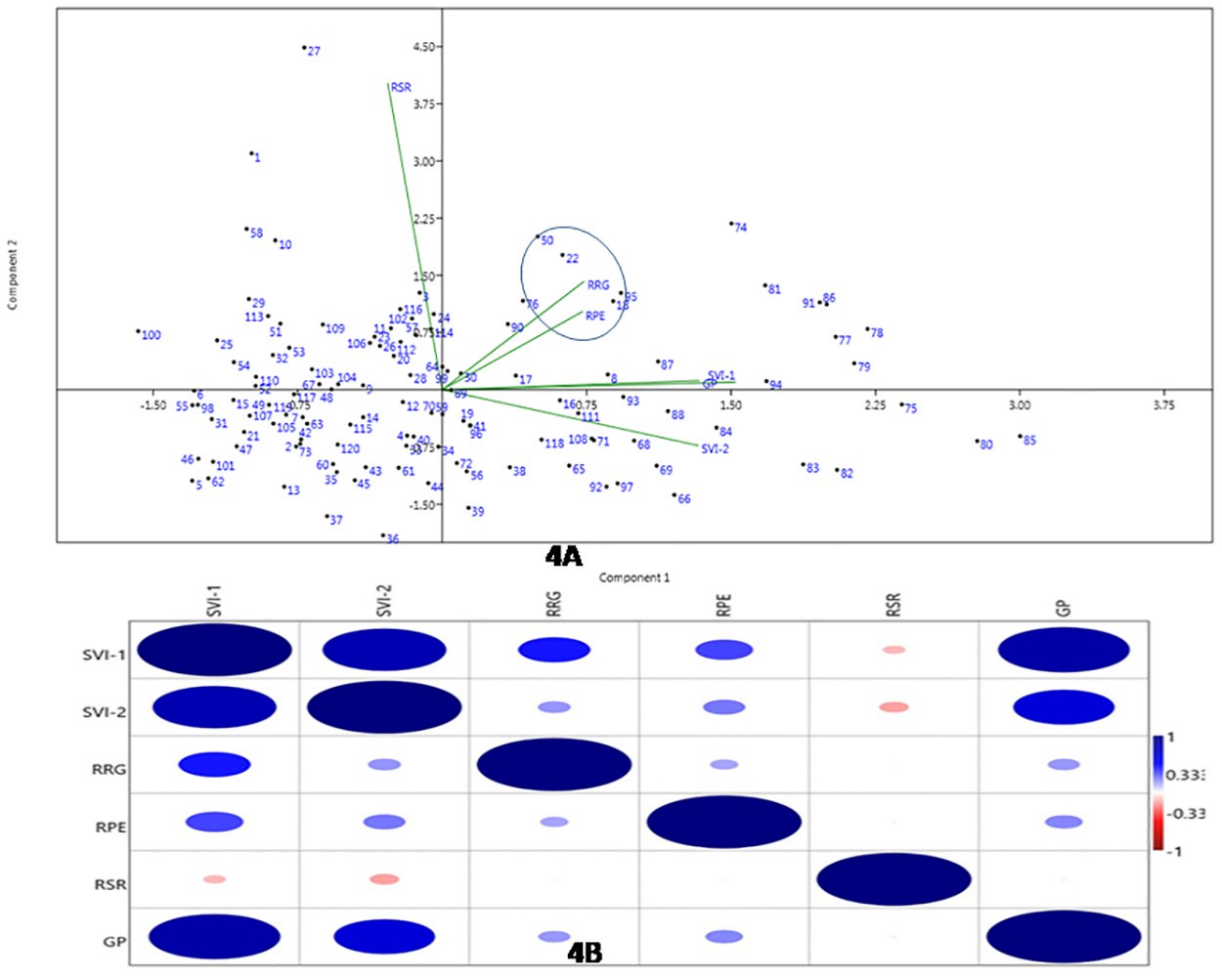
(A) Genotype-by-trait bi-plot diagram showing 120rice landraces in two PCs for 6 physiological traits (B) Heat map showing Pearson’s correlation coefficients for physiological traits. The dot numbers in the diagram depict the serial number of the germplasm line listed in Table 1. Significant correlations are colored either in red (negative correlation at 0.01 level) or blue hues (positive correlation at 0.01 level).

The scatter diagram was plotted by taking the first two principal components to generate genotype-by-trait biplot graph for the 6 physiological traits estimated from the 120 genotypes present in the panel (Fig 4A). The first and second principal components based on the correlation values showed 47.7.651 and 16.9of the total variability with eigen value of 2.86 and 1.02, respectively. RSR contributed maximum diversity among the 6 physiological parameters followed by SVII and SVI for the panel population based on the principal component analysis (Fig 4A). The scattering pattern of genotypes in the 4 quadrants indicated that genotypes containing high seedling stage growth parameters are placed in opposite direction of the quadrant 1 and II. Landraces with higher valuesof the physiological parameters have been encircled in the figure (Fig 4A). The top right (I^st^ quadrant) and bottom right (2^nd^ quadrant) accommodated majority of the genotypes containing high estimates of the studied physiological parameters. The 3^rd^ (bottom left) quadrant kept most of the moderate value containing landraces while the 4^th^ quadrant (top left) accommodated majority of low value carrying germplasm lines (Fig 4A).

The association among 6 physiological traits revealed a strong positive correlation (r≥0.7) of SV1 with SVI 2 and GP; SV2 with GP observed a moderate positive correlation (r:0.5–0.7). Weak positive correlation (r < 0.5) was observed for SVI with RRG and RPE; SV 2 with RRG, RPE and GP; RRG with RPE; GP with RRG and RPE. However, weak negative correlation was noticed for SVI II with RSR (Fig 4B).

### Genetic diversity parameters analysis

The constituted panel containing 120landraces from the original population of 274 landraces exhibited wide variation for the 6 physiological traits. The landraces were genotyped using 120SSR markers. The gene diversity, loci used for genetic diversity and other diversity related parameters are presented in S3 Table. A total of 544 marker alleles were obtained with average value of 4 alleles per locus. The range of alleles per locus varied from 2 to 7 per marker showing the highest number of alleles by RM493in the studied panel for the 6 physiological parameters. The average value of the major allele frequency of the parameters linked to the polymorphic markers was observed to be 0.561 which varied from 0.279 (RM8044) to0.925 (RM6054) (S3 Table). The range for PIC value was estimated to be from 0.137 (RM6054) to 0.787 (RM493) with mean value of 0.496. The observed average heterozygosity (Ho) in the population was 0.114 which varied from 0.00 to 0.958. The gene diversity (He) in the panel ranged from 0.1415 (RM6054) to 0.8126 (RM493) showing a mean value of 0.5545.

### Population genetic structure analysis

**Fig 5:**
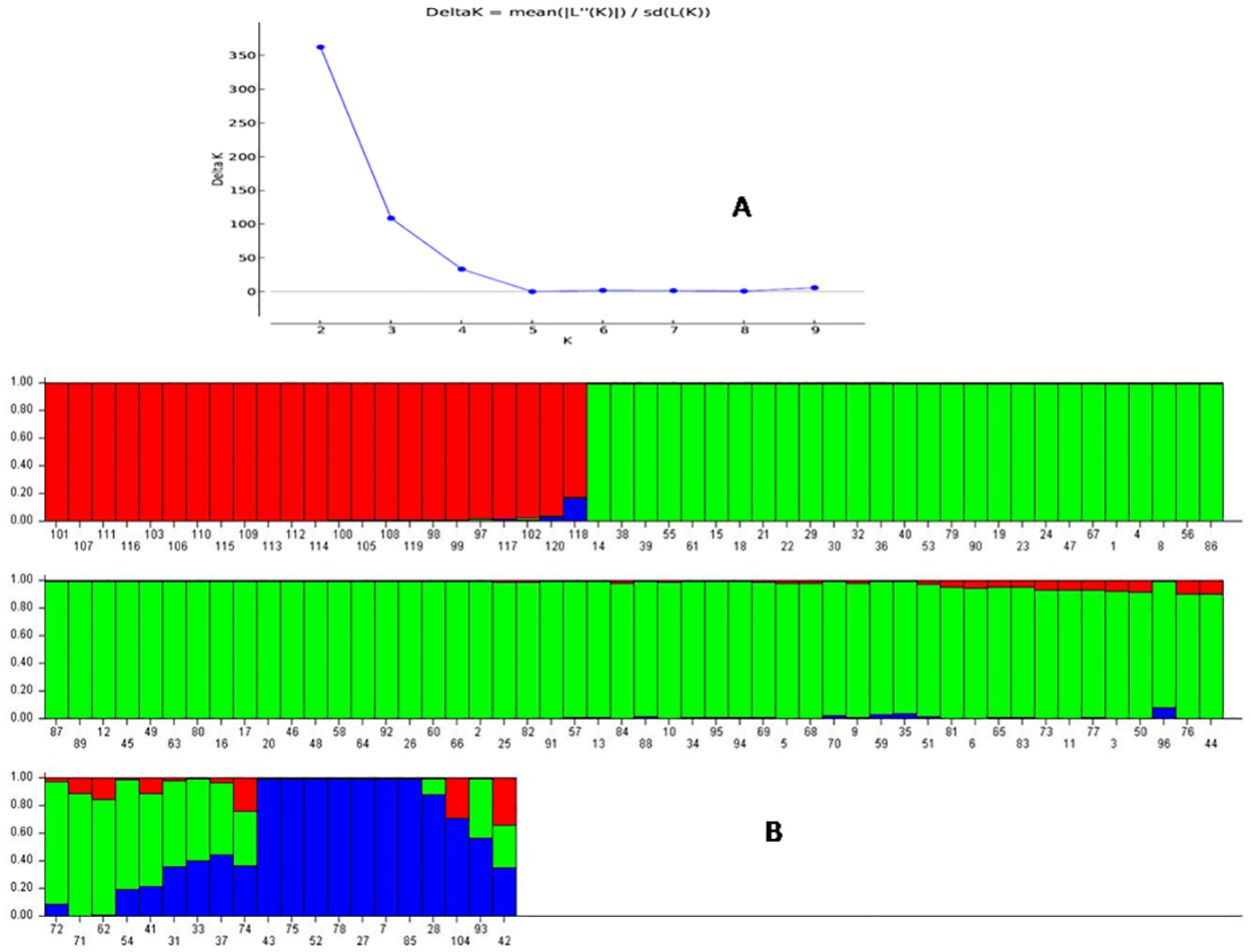
A) Graph of ΔK value, to the rate of change in the log probability of data between successive K values; B) Population structure for the 120 panel population based on membership probability fractions of individual genotypes at K = 3. The genotypes with the probability of ≥80% membership proportions were assigned as subgroups while others grouped as admixture group. The numbers in the diagram depict the serial number of the germplasm lines listed in Table 1.

The genotypes in the panel exhibiting variation for the studied seedling stage physiological parameters were evaluated for genetic structure by adopting the probable sub-populations (K) and selecting higher delta K-value estimated by STRUCTURE 2.3.6 software. The delta K value is related to the rate of change in the log probability of data between successive K values. It categorized the genotypes into two sub-populations with a high ΔK peak value of 362.4at K = 2 among the assumed K (S1 Fig). The proportions of genotypes in the inferred clusters were 0.277 and 0.723 in subpopulation 1 and subpopulation 2, respectively. The two subpopulations showed correspondence with the studied physiological parameters but presence of many unrelated landraces observed in the clusters. Hence, the next highest peak at the ΔK peak value of 108.8 was considered and the population was categorized into 3 subpopulations. The proportions of genotypes in the inferred clusters were 0.208, 0.689 and 0.103 for the sub-population 1, 2 and 3, respectively. The fixation index (Fst) values were 0.339, 0.166 and 0.370 for the sub-population 1, 2 and 3, respectively. The expected average distances or heterozygosity between individual in the clusters were 0.382, 0.428, and 0.393 in the sub-population 1, 2, and 3, respectively. The genotypes with ≥80% inferred ancestry value were categorized for that subpopulation (Table 1; Fig 5).

The seedling stage physiologicalparameters showed a relatively fair correspondence at K = 3 with the structure subpopulations presentin the panel population. Majority of the landraces showing high to very high seed vigour indices and root growth parameterswere present in the subpopulation SP1. Landraces such as AC44638, AC43663, AC44646, AC44604 and AC44598 present in this subpopulation are potential donor lines for seed vigour indices, germination % and root growth parameters. Majority of the members with poor to moderatein these parameters are found in the subpopulation SP2. Less priority may be given to the members of this subpopulation for use in the improvement of the target traits. The subpopulation 3 accommodated majority of the landraces showing moderate in seed vigour indices and root growth parameters. There is existence of a 4^th^ structure group for the admix landraceswherein majority of the moderate to high parameters carrying landraces are observed. The panel also showed a low alpha value (alpha = 0.0473) by the structure analysis at K = 3. Positively skewed leptokurtic distribution was observed for the mean alpha-value detected from the analysis. The distributions of Fst1, Fst2 and Fst3 subpopulations were symmetrically skewed showing a distinct variation in the distribution among the Fst values (S1 Fig).

### Molecular variance (AMOVA) and LD decay plot analysis

The closely related plants in a population are clustered into isolated groups and form various subpopulations. Genetic variations between and within the sub-populations at K = 3 were detected through analysis of molecular variance (AMOVA) (Table 2). The genetic variations obtained between and within at K = 3 was computed to be 18% among the populations, 63% among individuals and 19% variation within individuals of the panel population. Deviation from Hardy-Weinberg’s prediction was calculated from Wright’s F statistics estimates. Different parameters like uniformity of individual within the subpopulation (F_IS_) and individual within the total population (F_IT_) were estimated for differentiation of population. The F_IT_ and F_IS_ values of total population and within population based on 136 marker loci were 0.769 and 0.811, whereas F_ST_ was 0.181 between the two subpopulations. Fst is estimated to measure the population differentiation or the subpopulations within the total population. The Fst values of each sub-population and their distribution pattern showed a clear differentiation between the 3 sub-populations from each other (S2 Fig).

**Table 2:** Analysis of molecular variance (AMOVA) of the sub-populations present in the panel population at K = 3 for 6 physiological parameters in 120 rice germplasm lines.

Sources of variation	AMOVA for the four subpopulations at K = 3			
	df.	Mean sum of squares	Variance components	Percentage variation
Among populations	3	368.485	7.569	18%
Among individuals (accessions) within population	116	60.415	26.272	63%
Within individuals (accessions)	120	7.871	7.871	19%
Total	239		41.712	100%
F-Statistics	Value	*P*-value		
F_ST_	0.181	0.001		
F_IS_	0.769	0.001		
F_IT_	0.811	0.001		

**Fig 6:**
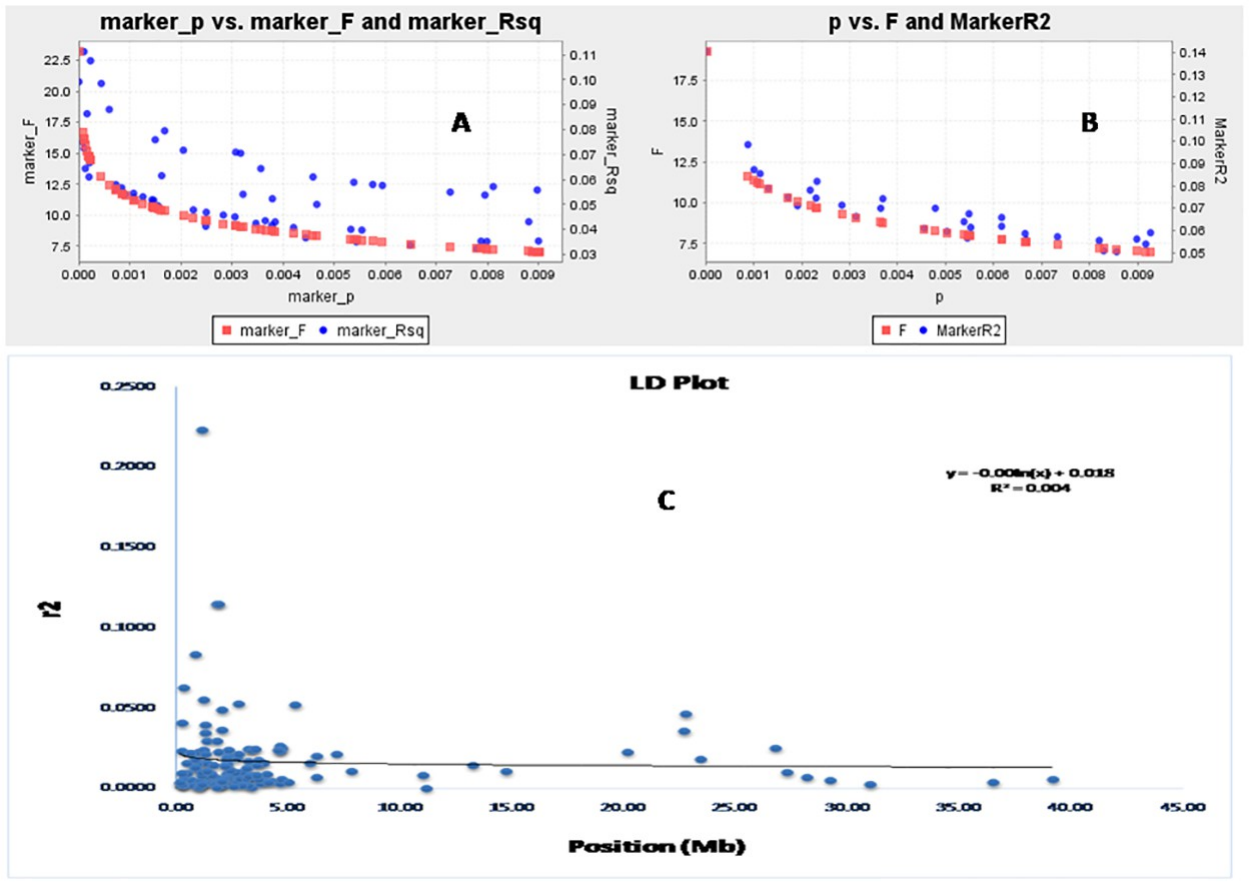
The marker ‘P’ versus marker ‘F’ and marker R^2^ detected using (A) GLM approach (B) MLM approach and (C)The physical distance (Mb) between pairs of loci on chromosomes against linkage disequilibrium (LD) decay (*r*^2^) curve plotted in rice. The decay started in million bp estimated by taking 95^th^ percentile of the distribution of r2 for all unlinked loci.

The association of markers alleles at different loci is successfully utilized for marker-trait association study. The LD decay rate is important factor for getting marker–trait association. The decay rate will facilitate the discovery of reliable markers associated with the physiological parameters and will facilitate the discovery of new genes or allelic variants controlling these traits. Syntenic*r*2 was used to plot the LD decay of the population against the physical distance in million base pair (Fig 6). Tightly linked markers have the highest *r*^2^ and average *r*^2^ rapidly decreases as linkage distance increases. There was a sharp decline in LD decay for the linked markers at 1–2 mega base pair and thereafter a very slow and gradual decay was noticed. Overall, it is clear that LD decay occur for the studied seedling stage physiological growth parameters. The genotypes with admixture type of ancestry values may be originated due to the LD decay of traits including the studied 6 traits. The trend is also seen in the marker ‘F’ versus marker ‘P’ and marker R^2^ (Fig 6) curve. The detected markers from this study indicated the strength of the markers for selection of the seedling stage physiological traits in rice.

### Genetic relatedness among the landracesby principal coordinates and cluster analyses

**Fig 7:**
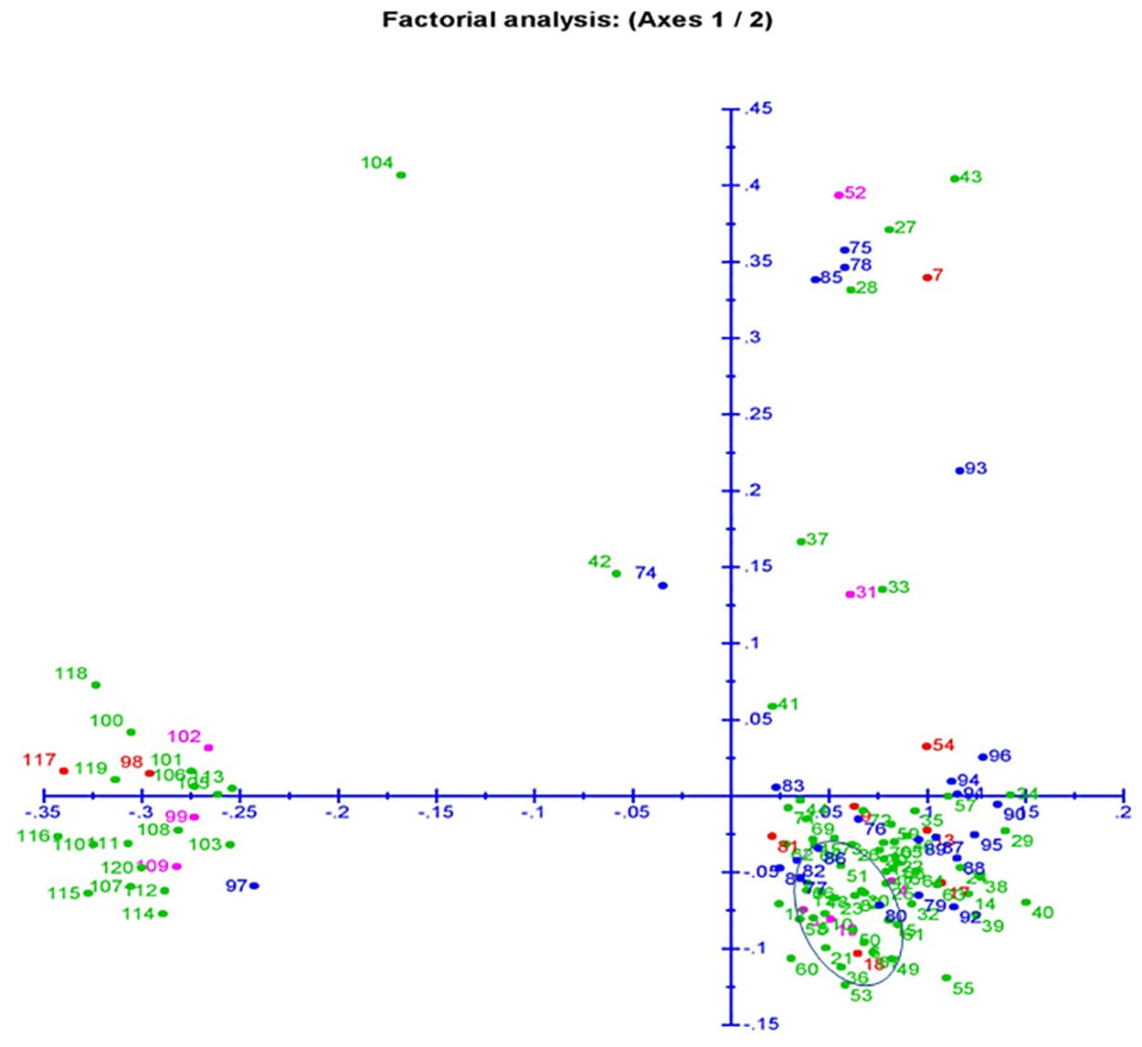
Principal coordinate analysis (PCoA) of 120landraces present in the panel population for 6 physiological traits using 136 molecular markers. The dot numbers in the figure represent the serial number of the genotypes enlisted in Table 1. The numbers are coloured on the basis of sub-populations obtained from structure analysis (SP1: Blue; SP2: pink; SP3: green and Admix: red).

The two dimensions diagram for principal coordinate analysis (PCoA) is drawn based on 136 markers genotyping data which grouped the landracesbased on the genetic relatedness among them (Fig 7). The component 1 accounted for 11.7% inertia and component 2 for 7.49% of total inertia. The panel containing landraceswere placed in various spots on the 4 quadrants which formed twomajor and two minor groups (Fig 7). Majority of the landraces showing higher values for the 6 physiological parameters are present in the quadrant 2 (right bottom). Almost all landraces in the subpopulation 1 and subpopulation 2 were with higher in seedling stage physiological traits present in this quadrant. The genotypes belonging to the 3^rd^ and 4^th^ quadrant showed poor to moderate in the studied seedling stage growth parameters. Majority of the admix type landraces depicted in red color are present in the quadrant 2. The best landraces with higher seedling stage physiological parameters were present in the 2^nd^ quadrantand encircled in the quadrant (Fig 7).

**Fig 8:**
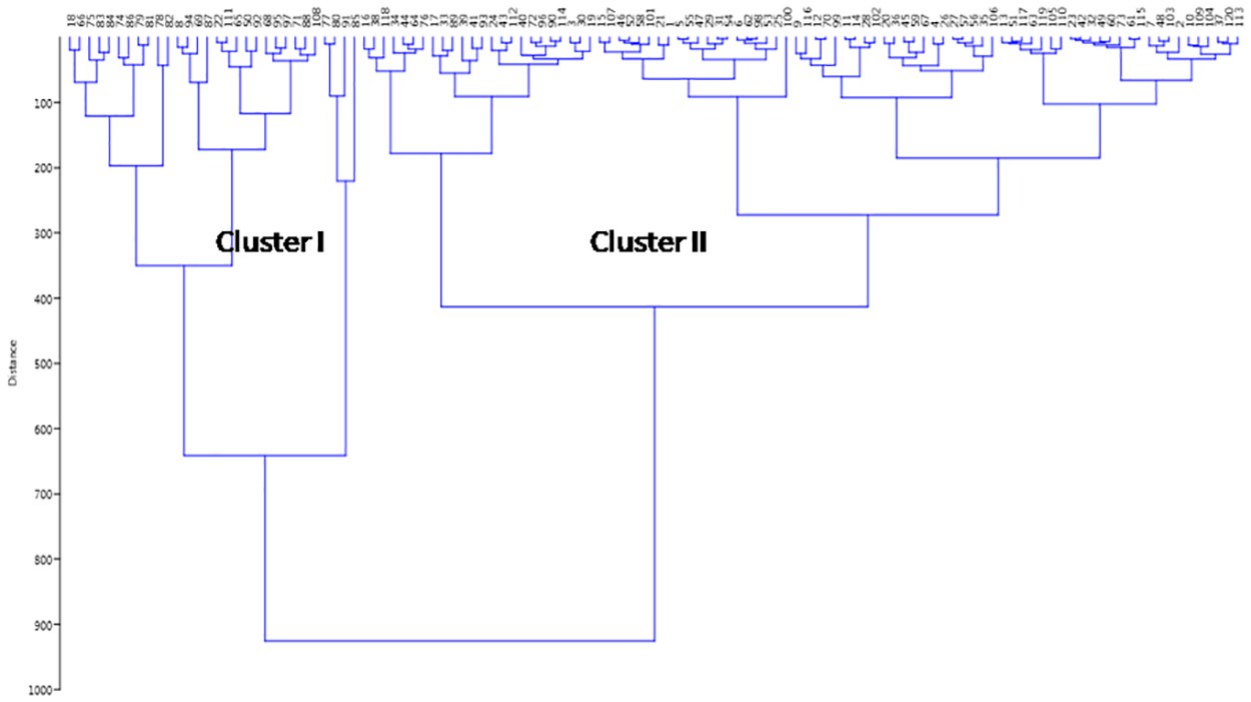
Wards’s clustering based on the estimates of 6 physiological traits for clustering of 120 germplasm lines.

The dendrogram is broadly classified into cluster I and cluster II based on the mean values of studied growth parameters (Fig 8). Cluster II is the biggest cluster which is classified into two sub clusters. Also, the cluster I is divided into two sub-clusters. Each sub-cluster is finally grouped into sub-sub clusters based on the presence of the growth parameter traits in the landraces. Majority of the germplasm lines present in the cluster I showed high or very high estimates of growth parameters in the landraces. Cluster II which accommodated 90 landraces while cluster I showed 30 genotypes. The germplasm lines present in the cluster II of the dendrogram were poor to moderate carrying parameters in it. The sub-clusters of Cluster II are finally grouped into sub-sub clusters based on the value of the growth parameters in the germplasm lines.

**Fig 9:**
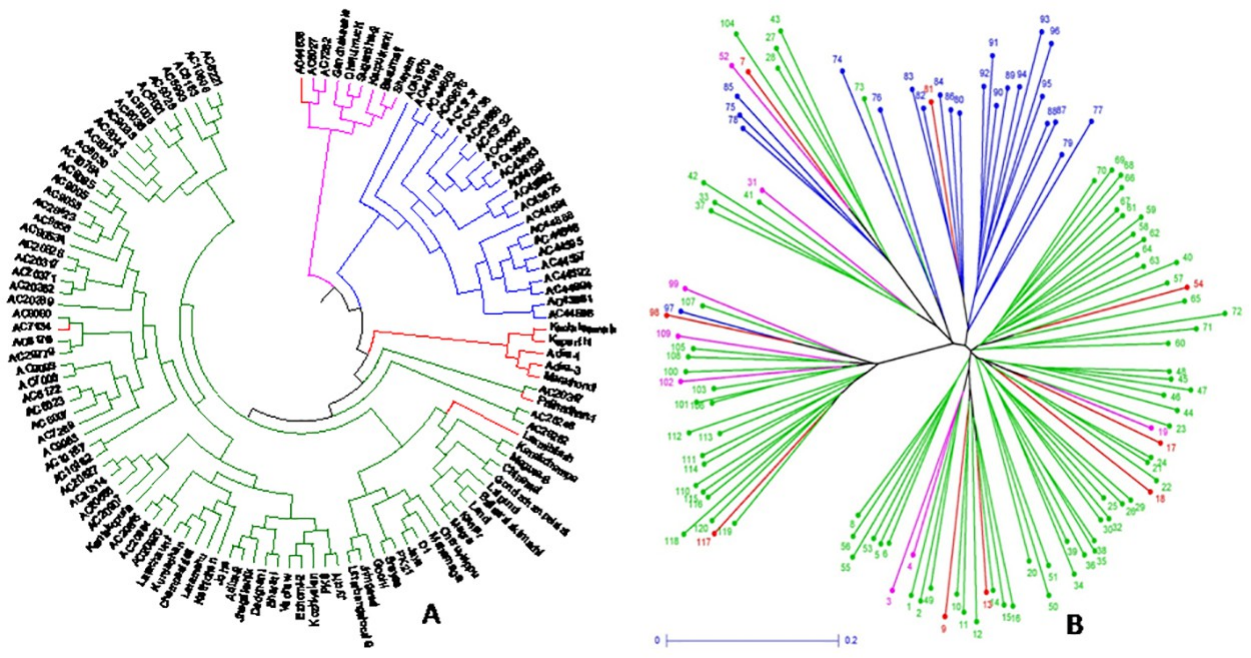
Tree constructed based on the genotyping results of 120 landraces using 136 SSR markers for depicting clustering pattern (A) UPGMA Unrooted tree (B) Neighbour-joining tree coloured on the basis of the sub-populations obtained from structure analysis at K = 3 (SP1: Blue; SP2: pink; SP3: green and Admix: red).

The UPGMA tree constructed based on the genotyping results using136 markers for the panel population that classified the germplasm lines into 4 groups including the admix type as in the case of PCoA. The colors of the 4 subpopulations are blue for SP1; green for SP2; pink for Sp3 and red for admix type (Fig 9A). Clusters SP_1_ was differentiated from SP_2_ by the presence of high estimates for the majority of the 6 studied physiological parameters. The landraces with poor and moderate in physiological parameters are in structure group 2. The germplasm lines with admix type of population are depicted in red color in the neighbour joining tree (Fig 9A). The phylogenetic tree is alsodrawn using unrooted tree. There is no common ancestor or node in this tree. The variations among the landraces are assessed from the distancefor each landrace depicted in the diagram (Fig 9B). The relationships among the landraces are determined using both the trees without considering the evolutionary time.

#### Association of marker alleles with the seedling stage physiological parameters in rice

**Fig 10:**
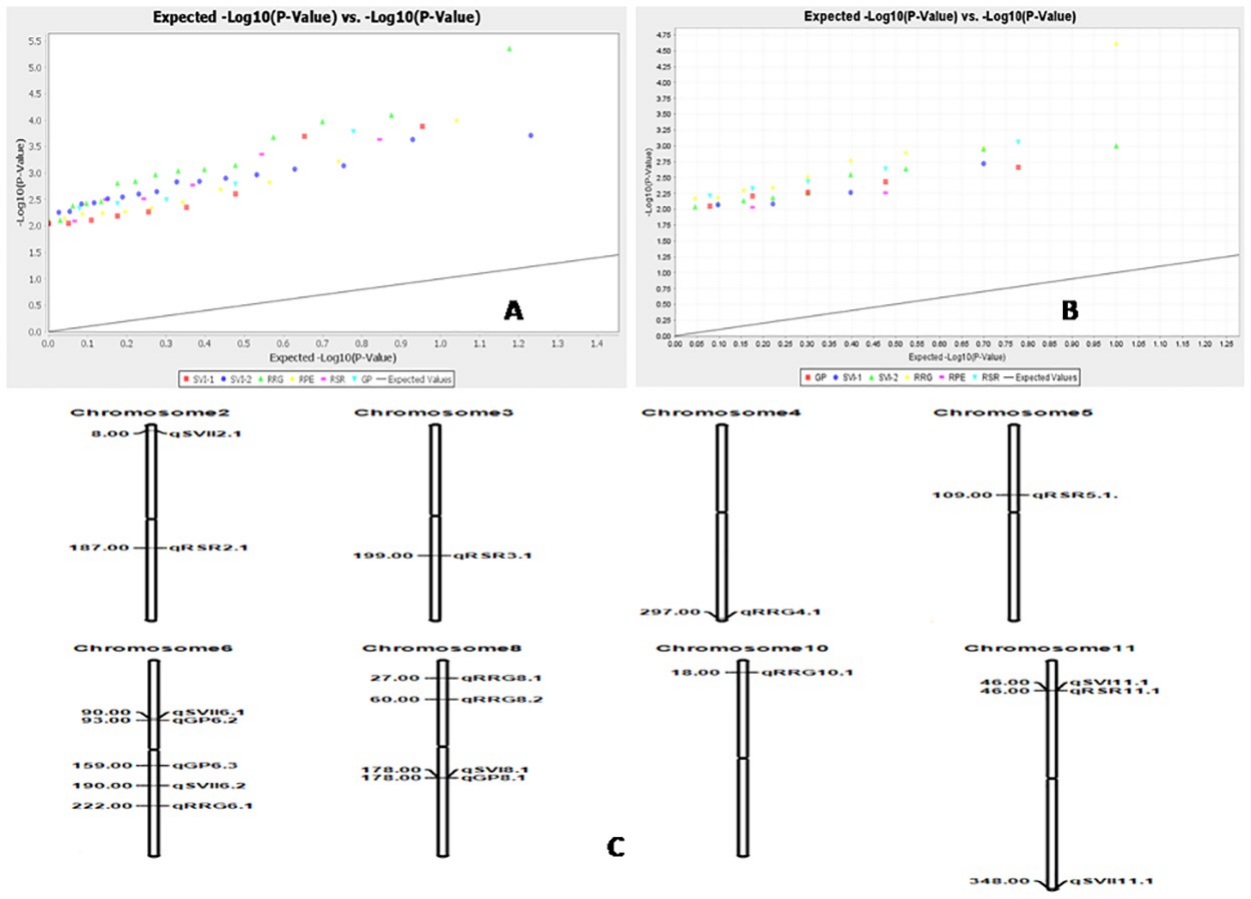
Distribution of marker-trait association and quantile–quantile (Q-Q) plot generated from Generalized Linear Model analysis for six antioxidant traits at (A) *p* < 0.05, (B) at *p* < 0.01 and (C) the positionsof the QTLs on the chromosomes for RSG, RGR, AGR and MGR detected byassociation mapping inrice.

Association of molecular markers with 6 physiological parameters was computed using Mixed Linear Model (MLM/ K+Q model) and Generalized Linear Model (GLM) by TASSEL 5 software. The marker-trait comparisons were subjected to filtration at less than 1% error i.e. 99% confidence (p<0.01). Five parameters showed significant associations with markers using both the models at p<0.01. A total of 39 and 32 significant marker-trait associations were detected by GLM and MLM, respectively at p<0.01 and markers R^2^ value >0.05. The marker R^2^ values computed by GLM approach was from 0.05001 to 0.1113 while the R^2^by MLM approach varied from 0.05042 to 0.14020 (S4 and S5 Tables). Significant marker-trait associations were detected for SVI-I with 2 markers; GPwith 3 markers; SVI-II and RSR with 4 markers;and RRG with 5 markers by both GML and MLM models at p<0.01 and considering markers R^2^ value at >0.05. Considering the marker r^2^ value of about 0.10 and above at p<0.01, marker RM223 exhibited associations with the parameter, RRG and RM405 with RSR analyzed by both the models (Table 3; S5 Table). The Q-Q plot also confirmed the association of these markers with the associated seed quality traits in rice (Fig 10).

**Table 3:** Marker alleles association with seed vigour index, root parameters and germination per cent in ricelandraces present in the panel population detected by both the models of GLM and MLM analyses at p<0.01.

SL.No	Traits	Marker	QTLs identified	Position (cM)	GLM				MLM			
					Marker_F	Marker_p	Marker_R^2^	q -value	Marker_F	Marker_p	Marker_R^2^	q- value
1	SVI-1	RM3701	*qSVI11*.*1*	46	14.73858	2.02E-04	0.06101	0.0039	8.02083	0.00545	0.05653	0.008364
2	SVI-1	RM502	*qSVI8*.*1*	178	15.66579	1.31E-04	0.06439	0.005657	7.21569	0.00829	0.05085	0.007171
3	SVI-2	RM13335	*qSVII2*.*1*	8	10.66622	0.00143	0.05185	0.001377	7.02387	0.00917	0.05385	0.00926
4	SVI-2	RM103	*qSVII6*.*1*	90	12.04771	7.29E-04	0.05793	0.001377	11.38112	0.00101	0.08726	0.008424
5	SVI-2	RM3	*qSVII6*.*2*	190	10.93544	0.00126	0.05304	0.001377	11.14453	0.00113	0.08545	0.00926
6	SVI-2	RM441	*qSVII11*.*1*	348	14.81491	1.95E-04	0.06973	0.0039	7.23687	0.0082	0.05549	0.00926
7	RRG	RM222	*qRRG10*.*1*	18	16.71582	8.04E-05	0.07491	0.003159	9.10484	0.00313	0.06625	0.007171
8	RRG	RM337	*qRRG8*.*1*	27	14.65199	2.10E-04	0.06669	0.0039	8.37216	0.00455	0.06092	0.007171
9	RRG	RM223	*qRRG8*.*2*	60	23.21457	4.42E-06	0.09917	0.001377	19.27957	2.51E-05	0.14029	0.00926
10	RRG	RM494	*qRRG6*.*1*	222	16.10333	1.07E-04	0.0725	0.001377	11.23597	0.00108	0.08176	0.008424
11	RRG	RM16686	*qRRG4*.*1*	297	11.58296	9.14E-04	0.05399	0.000287	10.85419	0.00131	0.07898	0.000853
12	RSR	RM3701	*RSR11*.*1*	46	9.14305	0.00307	0.07096	0.001377	8.27567	0.00478	0.06994	0.007171
13	RSR	RM405	*qRSR5*.*1*.	109	14.45053	2.31E-04	0.10758	0.003301	11.65994	8.81E-04	0.09854	0.007171
14	RSR	RM6641	*qRSR2*.*1*	187	13.09154	4.41E-04	0.09849	0.0039	8.78196	0.00369	0.07421	0.008424
15	RSR	RM168	*qRSR3*.*1*	199	10.34346	0.00168	0.07951	0.005657	7.7835	0.00617	0.06578	0.008424
16	GP	RM225	*qGP6*.*2*	93	10.41636	0.00162	0.06153	0.001377	8.80688	0.00365	0.06994	0.007171
17	GP	RM7179	*qGP6*.*3*	159	9.05346	0.00322	0.05406	0.002389	9.82798	0.00218	0.07805	0.008364
18	GP	RM502	*qGP8*.*1*	178	15.17228	1.65E-04	0.08637	0.0039	7.06356	0.00898	0.0561	0.008424

Two markers showed significant association with SVII detected by GLM and MLM models at p<0.01. Significant associations of markers RM3701 and RM502 with SVI were detected by both the models. Four markers namely RM13335, RM103, RM3 and RM441 located at 8, 90, 190 and 348 cM positions on chromosome 2, 6, 6 and 11, respectively were associated with the parameter, SVII. QTLs for germination % showed significant associations with RM225 on chromosome 6, RM7179 on chromosome 6 and RM502 on chromosome 8. The root-shoot parameter, RSR showed significant associations with markers, RM168, RM225, RM7179 and RM502. Markers *viz*., RM222, RM337, RM223, RM7179, RM494, RM16686 and RM243 showed significant association with the parameter, RRG. The parameter, RPE was detected to be significantly associated with 9 markers by GLM and by 2 markers in MLM analyses. But, no common marker was detected from the analysis by both the models. However, higher marker r2 and low p-values were shown by the marker RM25181, RM403 and RM309 analyzed by GLM model. Marker RM502 was strongly associated with parameters, SVI and GP. Marker RM7179 significantly associated with GP and RRG and hence both the traits are co-localized and controlled by segment near to 159 cM region on the chromosome 6. In addition, the traits controlled by SVI and RSR also associated with RM3701 (Table 3). The Q-Q plot also confirmed the associations of these markers with the estimated physiological parameters in rice **(**Fig 10).

## Discussion

Seed vigour improvement in rice is an important breeding objective mainly for the direct seeded rice. Genetics of this trait is complex in nature and hence directly and indirectly associated traits including the physiological traits are considered for the association study. Reports on association of markers with physiological traits are very less available in rice. The landraces included in this study were significantly different from each other for the 6 studied seedling stage physiological traits (Table 1). High PCV % and GCV % were estimated for SVI, SVII, RRG, RPE, RSR and GP indicating the usefulness of the landraces for the improvement programs. Higher magnitude of correlation coefficients of the few parameters will be helpful in deciding the traits associated with the seed vigour improvement and hence better for selection of progenies. The existenceof higher molecular diversity parameters in the population and also higher phenotypic variations for these 6 physiological traits indicated clear cut differentiations and classes in the studied population (Figs 5 and 7–9). The landraces used in this association study were collections from the states where existence of rich rice genetic diversity was reported in earlier studies [62–66]. Landraces from Jayapur region of Odisha state, the secondary centre of origin were also used in this experiment. However, the available diversity in the panel was from the collections made from 5 states of India, only. Few landraces viz., Champeisali, AC. 10187, AC. 3663, AC. 44638, AC. 44604, AC. 44646, AC. 44598, AC. 20362, AC. 9038, Kapanthi and Adira-2 showed presence of multiple seedling stage traits identified from the population (Table 1). Thus, inclusion of donor lines from this population will be effective for improvement of seed vigour. Use of diverse lines in many breeding and mapping studies for improvement traits are reported by many researchers in rice [1,67–77]. The panel population is broadly classified into cluster I and cluster II based on the mean values of studied growth parameters. Majority of the germplasm lines present in the cluster I showed presence of high or very high estimates of growth parameters in the landraces. The germplasm lines present in cluster II of the dendrogram were poor to moderate for the 6 studied seedling growth parameters in them. Three genetic structure groups carrying different Fst values were observed to be underlinkage disequilibrium for the studied traits in the population. Presence of many admix type landraces and low alpha value in the population provided clue for evolution of the traits from single source which formed different admix genotypes during the evolution process. Relatedness among the members of a structure subpopulation for the studied traits was proved from this study. The correspondence among the members of a structure group with seed vigour related traits were published by earlier workers [72,78]. Additionally, publications on phenotype of various traits and structure group relatedness have been published by many researchers [39,53,69,72–80].

Five traits for seedling stage physiological parameters were detected to be significantly associated with 18 SSR markers analyzed by both GLM and MLM approaches (Table 3). The markers associatedwith the parameters in this study were detected using both the models at p<0.01, low ‘p’ and higher marker r2 values are considered to be very robust and useful for seed vigour improvement program. Therefore the detected molecular markers namely RM3701 and RM502 with SVI; RM13335, RM103, RM3 and RM441 for SVI-II; RM225, RM7179 and RM502 for GP; RM168, RM225, RM7179 and RM502 for RSR; RM222, RM337, RM223, RM7179, RM494, RM16686 and RM243 with RRG are useful in molecular breeding for improvement of seed vigour trait in rice (Table 3). The Q-Q plot also confirmed the associations of these markers with various seed vigour influencing traits in rice (Fig 10). Molecular markers for seed vigour improvement were reported from many mapping studies [25,72,73,78,81].

Many QTLs controlling germination % in rice were reported by earlier researchers [17–23,26,28,30,31]. In our investigation, the markers RM225, RM7179 and RM502 were significantly associated with germination % and the QTLs were located near 92 cM and 159cM on the chromosome 6 and at 177 cM on chromosome 8, respectively. The two QTLs detected in our investigation were different from the genes reported by the above workers. These two QTLs detected for the trait in this mapping study were not reported in earlier studies and designated as *qGP6*.*2* and *qGP6*.*3*. The QTL detected by Jin et al. [31] on chromosome 8 is nearer to the QTL detected by us. Therefore, the QTL reported from earlier study, *qGP8*.*1* may be the detected QTL in our investigation. Anandan et al. [78] detected association of seed vigour index with marker, RM341 on chromosome 2. Diwan et al. [82] reported a QTL for seed vigour index on chromosome 2 within the marker interval RM174-RM145. In our investigation, markers RM13335 located on chromosome 2 showed significant associations with SVII by both the models. This QTL detected by us may be in the marker interval reported by Dewan et al. [82]. The QTL may be designed as *qSVII2*.*1* and validated usingthis mapping population. In addition, Diwan et al. [82] reported two QTLs on chromosome 6 present in the marker interval of RM3-RM162 and RM136-RM3 which controlled the seed vigour. We also detected two QTLs on chromosome 6, one associated with RM3 and other one near to it which are within the above two marker intervals. These QTLs may be designated as *qSVII6*.*1* and *qSVII6*.*2*which are validated in this mapping study. However, the QTL for the trait detected by us on chromosome8 near to 178 cM and chromosome 11 near to 46cM position for SV1 along with 348 cM position for SVII on chromosome 11 were not reported in earlier studies. Location ofvigour index reported in mapping studies of Liu et al.[22] and Zhang et al. [23] on the chromosome 11 were at different positions. These 3 QTLs are designated as *qSVI8*.*1*, *qSVI 11*.*1* and *qSVII 11*.*1*.

The trait relative root growth showed significant association with five markers namely RM222, RM337, RM223 RM494 and RM16686 present on the chromosomes 10, 8, 8, 6, and 4, respectively. According to the earlier mapping study, the QTLs for root length were reported on chromosome 8 [17] and chromosome 6 [15,33,34,35] but at different locations. The QTLs detected in this investigation for regulating RRG were designated as *qRRG10*.*1*, *qRRG8*.*1*, *qRRG8*.*2*, *qRRG6*.*1* and *qRRG4*.*1*. The trait, root shoot ratio showed significant association with four markers viz., RM6641, RM168, RM405 and RM3701 present on chromosome 2, 3, 5 and 11, respectively. As per earlier publications of Xu et al., Li et al. and Zhao et al. [25,36,37] the QTLs for the trait werealso reported but at different locations from the results of the present investigation. The detected QTLs were designated as *qRSR2*.*1*, *qRSR3*.*1* and *qRSR5*.*1*. However, the root length QTL reported by Sabar et al. [38] was located in the genomic region within the marker interval of RM202-RM229. In the present study, marker RM3701 is present within this interval and showed strong association with this trait. As the region reported by Sabar et al. [38] was for root related trait and the present mapping result is for root-shoot ratio, the reported QTL may be same one with the present detected QTL, *qRRL11*.*1*.

## Conclusion

Estimation of six seedling stage physiological parameters of a population containing 274 landraces showed wide genetic variations among the genotypes for the traits. Presence of linkage disequilibrium (LD) was detected in the panel population based on the fixation indices of the subpopulations. Moderate to high values of gene diversity, polymorphic information content (PIC) and other diversity parameters were estimated from the population by genotyping with 136 SSR markers. The population was classified into 3 genetic groups. The population was classified into subpopulations and each subpopulation showed relatedness for the 6 seedling stage physiological traits among the members in the subgroup. A total of 5 reported QTLs *viz*., *qGP8*.*1* for germination %; *qSVII2*.*1*, *qSVII6*.*1* and *qSVII6*.*2* for seed vigour index II, and *qRSR11*.*1* for root-shoot ratio were validated in this mapping population and will be useful for marker-assisted breeding. In addition, 13 QTLs regulating the physiological parameters such as *qSVI 11*.*1* for seed vigour index I; *qSVII 11*.*1*, and *qSVI12*.*1 for* seed vigour index II; *qRRG10*.*1*, *qRRG8*.*1*, *qRRG8*.*2*, *qRRG6*.*1* and *qRRG4*.*1* for rate of root growth; *qRSR2*.*1*, *qRSR3*.*1* and *qRSR5*.*1* for root-shoot ratio while *qGP6*.*2* and *qGP6*.*3* for germination % were identified using both the models of GLM and MLM analysis. Additionally, co-localization or co-inheritanceof QTLs, *qGP8*.*1 and qSVI8*.*1* for GP and SVI-1, *qGP6*.*2* and *qRRG6*.*1* for GP and RRG and SVI and *qSVI11*.*1* and *qRSR11*.*1 for SVI* and RSR were detected. The QTLs identified in this study will be useful for improvement of seed vigour trait in rice.

## Supporting information

S1 Fig Graph of ΔK value, to the rate of change in the log probability of data between successive K values; B) Population structure of the panel population containing 120 germplasm lines based on membership probability fractions of individual genotypes at K = 2. The genotypes with the probability of ≥80% membership proportions were assigned as subgroups while others grouped as admixture group. The numbers in the diagram depict the serial number of the germplasm lines listed in Table 1.
(TIF)

S2 Fig Distribution of (A) Alpha value of the panel (B) Fst values obtained for subpopulation 1; (C) Fst values obtained for subpopulation 2 and (D) Fst values obtained for subpopulation 3. (TIF)

S1 Table Mean estimates of seed vigour index, root growth parameters and germination per cent estimated from original population of 274 rice landraces. (DOCX)

S2 Table Markers information of the selected 136 SSR markers used for genotyping of 120 rice landraces. (DOCX)

S3 Table Estimation of genetic diversity parameters using 136 SSR markers loci in a panel population containing 120 rice landraces. (DOCX)

S4 Table Significant marker-trait associations detected for seed vigour index, root growth parameters and germination per cent by GLM approach at p<0.01. (DOCX)

S5 Table Significant marker-trait associations detected for seed vigour index, root parameters and germination per cent parameters by MLM approach at p<0.01. (DOCX)
