# Supplementary figures and images for: Unraveling the genomic regions controlling the seed vigour index, root growth parameters and germination per cent in rice
Source: PLoS One. 2022 Jul 26;17(7):e0267303. doi: 10.1371/journal.pone.0267303 (PMC9321372; doi:10.1371/journal.pone.0267303)

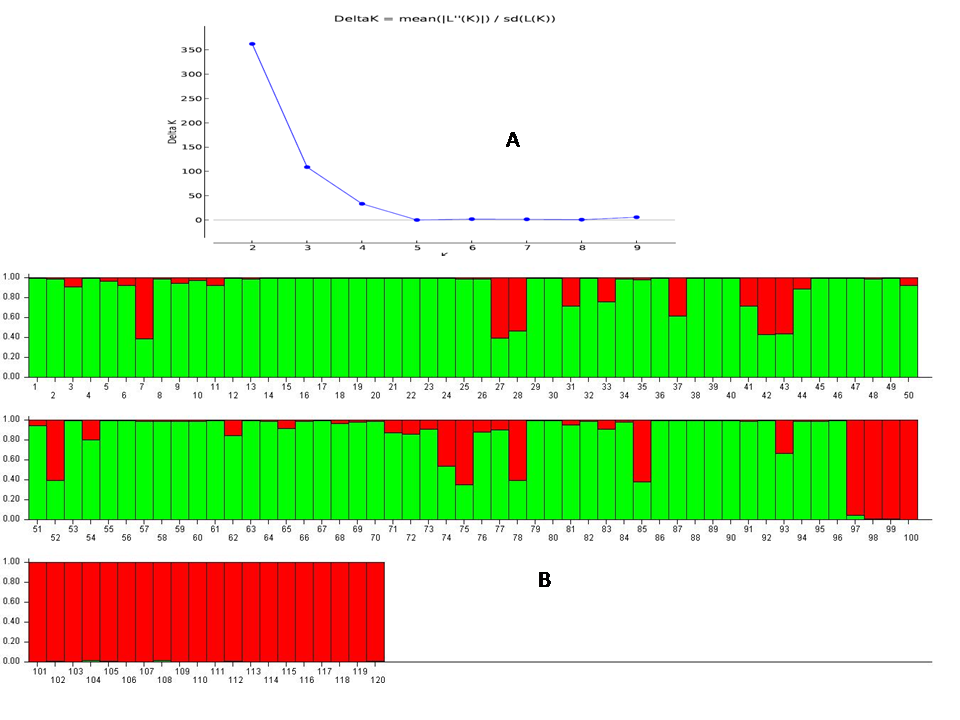

Supplement: S1 Fig — The genotypes with the probability of ≥80% membership proportions were assigned as subgroups while others grouped as admixture group. The numbers in the diagram depict the serial number of the germplasm lines listed in Table 1. (TIF) [file pone.0267303.s001.tif]

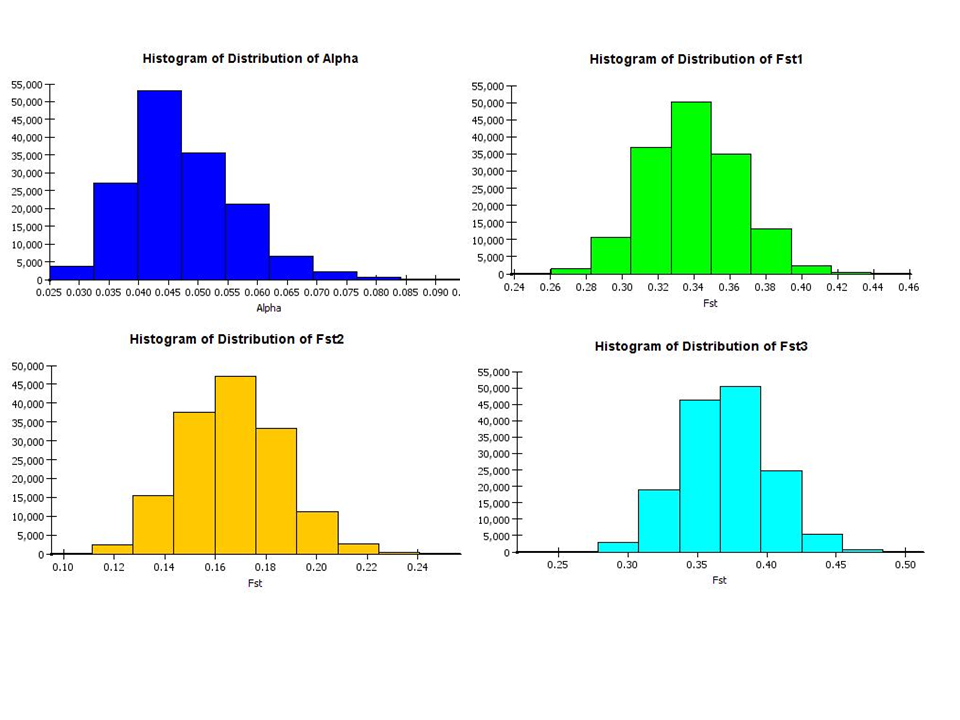

Supplement: S2 Fig — (TIF) [file pone.0267303.s002.tif]
