# Supplementary material for: Unraveling the genomic regions controlling the seed vigour index, root growth parameters and germination per cent in rice
Source: PLoS One. 2022 Jul 26;17(7):e0267303. doi: 10.1371/journal.pone.0267303 (PMC9321372; doi:10.1371/journal.pone.0267303)
Supplement: S1 Table — (DOCX) [file pone.0267303.s003.docx]

**Supplemental Table 1.** Mean estimates of seed vigour index, root growth parameters and germination per cent estimated from original population of 274 rice landraces.

| **Sl No.** | **Accession no./Vernacular name** | **SV-1** | **SV-2** | **RRG** | **RPE** | **RSR** | **GP** |
| --- | --- | --- | --- | --- | --- | --- | --- |
| 1 | *Magura-s | 411.180 | 0.165 | 0.632 | 0.480 | 0.760 | 33.000 |
| 2 | Magura manji | 929.940 | 0.297 | 0.483 | 0.520 | 0.914 | 66.000 |
| 3 | Kalakanhu | 525.300 | 0.240 | 0.245 | 0.240 | 1.067 | 60.000 |
| 4 | *Magra | 413.120 | 0.131 | 1.208 | 0.087 | 1.440 | 32.000 |
| 5 | Jata | 337.200 | 0.250 | 0.352 | 0.620 | 1.098 | 50.000 |
| 6 | *Kathidhan | 519.232 | 0.380 | 0.410 | 0.600 | 1.089 | 38.000 |
| 7 | Gargarh | 662.900 | 0.490 | 0.263 | 0.500 | 1.223 | 70.000 |
| 8 | Jhuli | 480.840 | 0.300 | 0.425 | 0.250 | 1.058 | 60.000 |
| 9 | *Karpurkanti | 605.150 | 0.282 | 0.398 | 0.543 | 0.815 | 65.000 |
| 10 | Rasuna | 368.000 | 0.400 | 0.352 | 0.400 | 0.791 | 50.000 |
| 11 | *Lal gundi | 320.760 | 0.252 | 0.238 | 0.672 | 0.632 | 36.000 |
| 12 | Latasal | 468.000 | 0.300 | 0.289 | 0.550 | 1.407 | 60.000 |
| 13 | Kasibaha | 687.400 | 0.420 | 0.341 | 0.320 | 1.154 | 70.000 |
| 14 | *Lata mahu | 335.700 | 0.180 | 0.818 | 0.830 | 0.701 | 30.000 |
| 15 | Rangi | 546.700 | 0.275 | 0.257 | 0.420 | 0.769 | 55.000 |
| 16 | Kalakadam | 512.850 | 0.260 | 0.412 | 0.280 | 0.831 | 65.000 |
| 17 | *Kantakaamala | 732.620 | 0.560 | 0.635 | 0.690 | 0.685 | 70.000 |
| 18 | Pankhi | 658.700 | 0.350 | 0.384 | 0.340 | 0.924 | 70.000 |
| 19 | *Kanak champa | 166.920 | 0.211 | 0.721 | 0.326 | 0.816 | 26.000 |
| 20 | Sodi | 547.200 | 0.240 | 0.261 | 0.420 | 0.744 | 60.000 |
| 21 | *Kunda dhan | 262.775 | 0.125 | 0.151 | 0.410 | 0.863 | 25.000 |
| 22 | Bhundi | 346.400 | 0.240 | 0.321 | 0.510 | 0.991 | 40.000 |
| 23 | *Landi | 313.424 | 0.266 | 0.539 | 0.265 | 1.022 | 38.000 |
| 24 | Daonra | 358.650 | 0.225 | 0.328 | 0.485 | 1.070 | 45.000 |
| 25 | *kapanthi | 1306.875 | 0.600 | 0.669 | 0.540 | 1.333 | 75.000 |
| 26 | Pasibutia | 430.100 | 0.220 | 0.321 | 0.512 | 0.970 | 55.000 |
| 27 | *Lata chaunri | 650.760 | 0.319 | 0.415 | 0.480 | 1.102 | 66.000 |
| 28 | Salkoiya | 439.500 | 0.400 | 0.287 | 0.556 | 1.123 | 50.000 |
| 29 | *Laxmi bilash | 293.500 | 0.150 | 0.284 | 0.580 | 0.866 | 25.000 |
| 30 | Guntha | 480.000 | 0.288 | 0.296 | 0.496 | 0.916 | 48.000 |
| 31 | Malata | 598.200 | 0.300 | 0.311 | 0.497 | 1.056 | 60.000 |
| 32 | *Kanta kapura | 907.200 | 0.540 | 0.438 | 0.683 | 0.710 | 72.000 |
| 33 | *Chinamal | 653.760 | 0.384 | 0.572 | 0.370 | 1.176 | 48.000 |
| 34 | Pindagadi | 426.500 | 0.200 | 0.287 | 0.501 | 0.827 | 50.000 |
| 35 | Baunsa gaja | 596.400 | 0.300 | 0.327 | 0.463 | 0.879 | 60.000 |
| 36 | *Gondia champeisiali | 947.050 | 0.780 | 0.498 | 0.720 | 0.833 | 65.000 |
| 37 | Palasphul | 653.900 | 0.390 | 0.296 | 0.384 | 0.946 | 65.000 |
| 38 | *Balisara lakti machi -k | 592.327 | 0.205 | 1.178 | 0.480 | 1.068 | 41.000 |
| 39 | Matia | 417.600 | 0.180 | 0.319 | 0.402 | 0.871 | 45.000 |
| 40 | Sarada | 291.550 | 0.175 | 0.283 | 0.445 | 0.791 | 35.000 |
| 41 | *Champaeisiali | 990.600 | 0.420 | 1.007 | 0.750 | 1.306 | 60.000 |
| 42 | Ladu | 370.800 | 0.200 | 0.214 | 0.432 | 0.813 | 40.000 |
| 43 | *Kaniar | 916.080 | 0.616 | 0.407 | 0.560 | 0.648 | 66.000 |
| 44 | Madhabi | 425.880 | 0.270 | 0.235 | 0.425 | 0.855 | 45.000 |
| 45 | AC9011 | 340.557 | 0.412 | 0.821 | 0.130 | 0.970 | 22.000 |
| 46 | *AC9050 | 245.665 | 0.490 | 0.213 | 0.248 | 1.812 | 35.000 |
| 47 | AC9102 | 241.440 | 0.636 | 0.737 | 0.310 | 2.144 | 24.000 |
| 48 | AC9119A | 177.360 | 0.732 | 0.343 | 0.320 | 1.213 | 24.000 |
| 49 | *AC9093 | 417.312 | 1.176 | 0.451 | 0.323 | 1.577 | 42.000 |
| 50 | AC9008 | 456.750 | 1.125 | 0.365 | 0.318 | 1.246 | 45.000 |
| 51 | AC9010 | 390.000 | 0.720 | 0.421 | 0.225 | 0.864 | 40.000 |
| 52 | *AC9043 | 800.124 | 0.884 | 0.273 | 0.139 | 0.739 | 52.000 |
| 53 | AC9016 | 399.500 | 1.050 | 0.389 | 0.320 | 1.201 | 50.000 |
| 54 | AC9019 | 379.080 | 0.760 | 0.356 | 0.250 | 1.069 | 40.000 |
| 55 | *AC9044A | 678.680 | 0.640 | 1.215 | 0.085 | 0.883 | 40.000 |
| 56 | AC9022 | 406.525 | 0.595 | 0.387 | 0.180 | 0.829 | 35.000 |
| 57 | AC9023 | 333.600 | 0.440 | 0.426 | 0.240 | 0.706 | 40.000 |
| 58 | AC9025 | 576.125 | 0.440 | 0.453 | 0.230 | 1.003 | 55.000 |
| 59 | *AC9005 | 876.120 | 0.637 | 0.811 | 0.293 | 1.099 | 49.000 |
| 60 | AC9059 | 430.800 | 0.480 | 0.367 | 0.190 | 1.326 | 40.000 |
| 61 | *AC9076A | 173.988 | 0.192 | 0.551 | 0.582 | 1.691 | 12.000 |
| 62 | AC9063 | 294.300 | 0.420 | 0.486 | 0.110 | 1.142 | 30.000 |
| 63 | AC9076 | 574.200 | 0.880 | 0.397 | 0.120 | 0.676 | 55.000 |
| 64 | *AC9030 | 557.460 | 0.912 | 0.521 | 0.106 | 0.598 | 38.000 |
| 65 | AC9081 | 442.800 | 0.810 | 0.421 | 0.080 | 0.889 | 45.000 |
| 66 | *AC9058 | 358.040 | 0.920 | 0.245 | 0.420 | 1.476 | 40.000 |
| 67 | AC9086 | 418.500 | 0.950 | 0.658 | 0.130 | 0.726 | 50.000 |
| 68 | AC9095 | 284.700 | 0.510 | 0.532 | 0.210 | 0.797 | 30.000 |
| 69 | *AC9028 | 491.840 | 0.736 | 0.918 | 0.062 | 0.817 | 32.000 |
| 70 | AC9097 | 564.000 | 0.550 | 0.471 | 0.180 | 1.004 | 50.000 |
| 71 | *AC9065 | 487.710 | 1.170 | 0.271 | 0.421 | 1.505 | 45.000 |
| 72 | AC9099 | 411.200 | 0.320 | 0.329 | 0.170 | 1.073 | 40.000 |
| 73 | AC9118 | 512.500 | 0.600 | 0.421 | 0.190 | 0.821 | 50.000 |
| 74 | *AC9063 | 329.596 | 0.850 | 0.373 | 0.130 | 1.760 | 34.000 |
| 75 | AC9155 | 370.400 | 0.560 | 0.432 | 0.220 | 0.630 | 40.000 |
| 76 | AC9157 | 319.500 | 0.480 | 0.386 | 0.230 | 0.784 | 30.000 |
| 77 | AC9232 | 504.500 | 0.550 | 0.412 | 0.180 | 1.080 | 50.000 |
| 78 | *AC9006 | 734.988 | 0.644 | 0.741 | 0.165 | 0.859 | 46.000 |
| 79 | AC9241 | 396.550 | 0.420 | 0.389 | 0.160 | 1.175 | 35.000 |
| 80 | *AC9053A | 105.280 | 0.080 | 0.140 | 0.270 | 1.251 | 16.000 |
| 81 | AC9248 | 445.200 | 0.440 | 0.468 | 0.223 | 0.970 | 40.000 |
| 82 | AC9268 | 597.850 | 0.440 | 0.154 | 0.220 | 1.082 | 55.000 |
| 83 | *AC9021 | 868.840 | 0.580 | 0.263 | 0.240 | 0.906 | 58.000 |
| 84 | AC9273 | 604.800 | 0.720 | 0.365 | 0.230 | 1.118 | 60.000 |
| 85 | *AC9038 | 873.496 | 1.040 | 0.911 | 0.220 | 0.865 | 52.000 |
| 86 | AC9278 | 376.000 | 0.440 | 0.378 | 0.180 | 1.207 | 40.000 |
| 87 | AC9077 | 350.280 | 0.490 | 0.418 | 0.150 | 0.951 | 35.000 |
| 88 | AC9094 | 436.800 | 0.520 | 0.435 | 0.140 | 0.926 | 40.000 |
| 89 | AC9096 | 347.700 | 0.330 | 0.428 | 0.160 | 1.119 | 30.000 |
| 90 | *AC9035 | 388.800 | 0.550 | 0.903 | 0.150 | 0.576 | 25.000 |
| 91 | AC9122 | 537.000 | 0.600 | 0.388 | 0.110 | 1.196 | 50.000 |
| 92 | *AC9090 | 464.100 | 1.131 | 0.481 | 0.430 | 2.208 | 39.000 |
| 93 | AC9127 | 529.650 | 0.605 | 0.357 | 0.120 | 1.071 | 55.000 |
| 94 | *Pk-21 | 1113.770 | 0.588 | 0.778 | 0.267 | 0.928 | 98.000 |
| 95 | *Mahamaga | 258.000 | 0.172 | 0.445 | 0.194 | 0.920 | 43.000 |
| 96 | Umamata | 212.160 | 0.091 | 0.540 | 0.210 | 1.061 | 26.000 |
| 97 | * D1 | 188.930 | 0.131 | 0.362 | 0.205 | 0.988 | 35.000 |
| 98 | Uma | 451.500 | 0.350 | 0.258 | 0.140 | 1.232 | 70.000 |
| 99 | M-10 | 369.180 | 0.120 | 0.254 | 0.140 | 0.726 | 60.000 |
| 100 | *Ezhoml-2 | 523.200 | 0.300 | 0.238 | 0.052 | 0.995 | 75.000 |
| 101 | Nambial Amber | 340.080 | 0.160 | 0.326 | 0.180 | 1.000 | 40.000 |
| 102 | Aiswarya | 326.250 | 0.150 | 0.289 | 0.140 | 1.004 | 50.000 |
| 103 | *Kozhivalan | 600.576 | 0.363 | 0.102 | 0.335 | 1.048 | 68.000 |
| 104 | Kanchan | 474.960 | 0.120 | 0.318 | 0.220 | 1.152 | 60.000 |
| 105 | *Cheruvirippu | 406.280 | 0.336 | 0.315 | 0.281 | 0.977 | 56.000 |
| 106 | Kunje Kunje | 453.310 | 0.275 | 0.452 | 0.160 | 0.809 | 55.000 |
| 107 | Harsha | 555.240 | 0.420 | 0.269 | 0.180 | 0.515 | 70.000 |
| 108 | *Adira-3 Pallakad | 619.372 | 0.364 | 0.539 | 0.217 | 1.171 | 52.000 |
| 109 | Kanjana | 509.080 | 0.325 | 0.462 | 0.220 | 0.835 | 65.000 |
| 110 | Adhuthurai | 629.940 | 0.420 | 0.358 | 0.180 | 0.682 | 60.000 |
| 111 | *Gandhakasala | 401.400 | 0.240 | 0.740 | 0.145 | 1.351 | 60.000 |
| 112 | Ambeponar | 372.680 | 0.200 | 0.351 | 0.160 | 1.528 | 40.000 |
| 113 | * Sreyas | 231.712 | 0.123 | 0.672 | 0.195 | 1.138 | 32.000 |
| 114 | *Jaya | 446.208 | 0.299 | 0.529 | 0.244 | 1.317 | 64.000 |
| 115 | Triveni | 547.330 | 0.280 | 0.456 | 0.190 | 1.191 | 70.000 |
| 116 | *PK6 | 259.578 | 0.219 | 0.463 | 0.180 | 0.652 | 46.000 |
| 117 | Chengarama | 479.440 | 0.390 | 0.263 | 0.210 | 0.735 | 65.000 |
| 118 | *Adira-1 Pallakad | 381.024 | 0.214 | 0.235 | 0.408 | 0.918 | 28.000 |
| 119 | *Jyothi | 190.066 | 0.116 | 0.418 | 0.184 | 1.095 | 29.000 |
| 120 | Kuttosam | 275.160 | 0.200 | 0.328 | 0.180 | 0.728 | 40.000 |
| 121 | Thavalakkaman | 308.560 | 0.200 | 0.337 | 0.200 | 0.813 | 40.000 |
| 122 | *Marathondi | 262.444 | 0.111 | 0.387 | 0.410 | 0.687 | 26.000 |
| 123 | Champan mata | 533.610 | 0.330 | 0.346 | 0.180 | 0.893 | 55.000 |
| 124 | Kalladiyaran | 615.540 | 0.240 | 0.298 | 0.210 | 0.993 | 60.000 |
| 125 | *Adira-2 Pallakad | 1293.870 | 0.581 | 0.748 | 0.425 | 1.066 | 85.000 |
| 126 | Onam | 602.562 | 0.290 | 0.311 | 0.140 | 0.916 | 58.000 |
| 127 | *Vachaw | 581.872 | 0.601 | 0.396 | 0.212 | 1.282 | 82.000 |
| 128 | Nooravella | 483.700 | 0.250 | 0.302 | 0.180 | 1.121 | 50.000 |
| 129 | Airweraga | 268.508 | 0.133 | 0.238 | 0.490 | 0.773 | 38.000 |
| 130 | Chetadi | 386.568 | 0.252 | 0.412 | 0.200 | 1.128 | 42.000 |
| 131 | Amballavalaya | 351.082 | 0.190 | 0.405 | 0.220 | 1.240 | 38.000 |
| 132 | AC20117 | 457.380 | 0.504 | 0.580 | 0.400 | 0.754 | 42.000 |
| 133 | *AC20770 | 692.752 | 0.348 | 0.293 | 0.253 | 0.315 | 58.000 |
| 134 | AC20436 | 616.560 | 0.305 | 0.973 | 0.380 | 0.950 | 42.000 |
| 135 | *AC20423 | 502.384 | 0.408 | 1.085 | 0.208 | 1.058 | 34.000 |
| 136 | AC20604 | 487.200 | 0.260 | 0.997 | 0.390 | 0.750 | 40.000 |
| 137 | *AC20614 | 390.474 | 0.287 | 0.318 | 0.430 | 0.971 | 42.000 |
| 138 | *AC20686 | 188.388 | 0.054 | 0.152 | 0.207 | 0.841 | 18.000 |
| 139 | AC20690 | 437.400 | 0.153 | 0.247 | 0.210 | 0.960 | 36.000 |
| 140 | *AC20845 | 635.626 | 0.645 | 0.532 | 0.136 | 0.806 | 43.000 |
| 141 | *AC20627 | 481.194 | 0.210 | 0.229 | 0.386 | 0.662 | 42.000 |
| 142 | AC20071 | 621.060 | 0.660 | 0.645 | 0.240 | 0.836 | 55.000 |
| 143 | *AC20664 | 138.780 | 0.108 | 0.225 | 0.432 | 0.928 | 18.000 |
| 144 | *AC20907 | 306.480 | 0.165 | 0.271 | 0.412 | 0.838 | 30.000 |
| 145 | AC20081 | 513.750 | 0.400 | 0.550 | 0.350 | 1.119 | 50.000 |
| 146 | *AC20246 | 508.608 | 0.456 | 0.409 | 0.570 | 1.070 | 48.000 |
| 147 | AC20095 | 454.725 | 0.495 | 0.540 | 0.380 | 0.932 | 45.000 |
| 148 | AC20113 | 492.000 | 0.480 | 0.480 | 0.410 | 0.994 | 48.000 |
| 149 | *AC20282 | 1104.932 | 0.771 | 0.895 | 0.400 | 0.777 | 68.000 |
| 150 | *AC20328 | 1218.168 | 1.134 | 0.562 | 0.360 | 0.744 | 84.000 |
| 151 | AC20119 | 445.200 | 0.320 | 0.565 | 0.120 | 0.890 | 40.000 |
| 152 | *AC20371 | 1329.093 | 1.211 | 0.333 | 0.300 | 0.679 | 92.000 |
| 153 | AC20121 | 385.175 | 0.245 | 0.614 | 0.280 | 0.801 | 35.000 |
| 154 | *AC20347 | 333.270 | 0.135 | 0.558 | 0.400 | 0.585 | 30.000 |
| 155 | AC20128 | 421.200 | 0.240 | 0.345 | 0.320 | 0.943 | 40.000 |
| 156 | *AC20362 | 1126.930 | 0.893 | 0.395 | 0.562 | 0.718 | 85.000 |
| 157 | *AC20389 | 1008.764 | 1.066 | 0.508 | 0.286 | 0.801 | 82.000 |
| 158 | AC20136 | 441.450 | 0.405 | 0.452 | 0.300 | 0.912 | 45.000 |
| 159 | *AC20317 | 590.660 | 0.840 | 0.351 | 0.338 | 0.825 | 70.000 |
| 160 | *AC20920 | 753.246 | 0.580 | 1.118 | 0.280 | 0.508 | 58.000 |
| 161 | AC20137 | 533.500 | 0.500 | 0.478 | 0.280 | 0.954 | 50.000 |
| 162 | AC5946 | 311.220 | 0.126 | 0.378 | 0.210 | 0.799 | 42.000 |
| 163 | *AC10187 | 1049.040 | 0.480 | 1.853 | 0.228 | 1.693 | 80.000 |
| 164 | AC6006 | 119.520 | 0.144 | 0.823 | 0.260 | 1.688 | 18.000 |
| 165 | *AC6023 | 147.664 | 0.160 | 1.078 | 0.400 | 1.462 | 16.000 |
| 166 | AC6617 | 124.640 | 0.480 | 0.633 | 0.180 | 0.972 | 16.000 |
| 167 | *AC7008 | 131.680 | 0.088 | 0.669 | 0.118 | 1.060 | 16.000 |
| 168 | AC7009 | 117.040 | 0.053 | 0.563 | 0.080 | 0.949 | 14.000 |
| 169 | AC7124 | 65.040 | 0.051 | 0.290 | 0.090 | 1.297 | 12.000 |
| 170 | AC7204 | 113.920 | 0.200 | 0.657 | 0.430 | 1.070 | 16.000 |
| 171 | *AC7269 | 123.260 | 0.160 | 0.612 | 0.673 | 1.145 | 20.000 |
| 172 | AC10333 | 198.600 | 0.110 | 0.377 | 0.080 | 1.115 | 30.000 |
| 173 | AC10438 | 88.000 | 0.072 | 0.497 | 0.110 | 1.381 | 16.000 |
| 174 | *AC10608 | 320.340 | 0.176 | 0.440 | 0.120 | 1.092 | 38.000 |
| 175 | AC5757 | 370.020 | 0.168 | 0.458 | 0.140 | 1.073 | 42.000 |
| 176 | AC5768 | 329.200 | 0.120 | 0.521 | 0.150 | 1.138 | 40.000 |
| 177 | AC5813 | 292.600 | 0.190 | 0.468 | 0.210 | 0.869 | 38.000 |
| 178 | AC5828 | 311.500 | 0.210 | 0.523 | 0.220 | 1.438 | 35.000 |
| 179 | AC5832 | 366.300 | 0.225 | 0.356 | 0.180 | 1.505 | 45.000 |
| 180 | AC5840 | 341.550 | 0.220 | 0.412 | 0.160 | 1.426 | 55.000 |
| 181 | *AC6183 | 475.798 | 0.209 | 1.638 | 0.360 | 2.361 | 38.000 |
| 182 | AC5951 | 294.800 | 0.200 | 0.402 | 0.180 | 1.042 | 40.000 |
| 183 | AC5965 | 318.060 | 0.266 | 0.386 | 0.190 | 1.174 | 38.000 |
| 184 | *AC6027 | 150.040 | 0.120 | 0.561 | 0.176 | 1.051 | 20.000 |
| 185 | *AC10162 | 274.800 | 0.150 | 1.098 | 0.368 | 1.245 | 30.000 |
| 186 | AC6148 | 250.320 | 0.140 | 0.256 | 0.230 | 1.104 | 28.000 |
| 187 | *AC7282 | 183.920 | 0.110 | 0.921 | 0.380 | 1.005 | 22.000 |
| 188 | AC6156 | 266.700 | 0.180 | 0.425 | 0.240 | 1.735 | 30.000 |
| 189 | AC6235 | 255.680 | 0.160 | 0.586 | 0.140 | 1.378 | 32.000 |
| 190 | *AC6221 | 491.640 | 0.374 | 0.619 | 0.180 | 1.310 | 68.000 |
| 191 | AC6237 | 162.600 | 0.080 | 0.478 | 0.150 | 1.853 | 20.000 |
| 192 | AC6562 | 297.150 | 0.175 | 0.598 | 0.160 | 0.834 | 35.000 |
| 193 | AC6571 | 272.340 | 0.136 | 0.514 | 0.200 | 0.885 | 34.000 |
| 194 | *AC-5993 | 99.000 | 0.072 | 0.249 | 0.349 | 1.282 | 18.000 |
| 195 | AC6633 | 320.340 | 0.114 | 0.486 | 0.180 | 1.190 | 38.000 |
| 196 | *AC7134 | 156.860 | 0.110 | 0.681 | 0.288 | 1.140 | 22.000 |
| 197 | AC7031 | 353.200 | 0.160 | 0.385 | 0.200 | 1.393 | 40.000 |
| 198 | AC7041 | 454.860 | 0.210 | 0.547 | 0.220 | 0.811 | 42.000 |
| 199 | *AC6170 | 521.645 | 0.184 | 0.452 | 0.208 | 1.151 | 85.000 |
| 200 | AC7073 | 427.200 | 0.288 | 0.512 | 0.180 | 1.084 | 48.000 |
| 201 | *AC6007 | 310.080 | 0.210 | 0.962 | 0.235 | 1.256 | 30.000 |
| 202 | AC7089 | 272.100 | 0.150 | 0.485 | 0.200 | 1.296 | 30.000 |
| 203 | AC7093 | 398.500 | 0.400 | 0.496 | 0.180 | 0.934 | 50.000 |
| 204 | *AC6172 | 668.460 | 0.478 | 0.623 | 0.230 | 0.998 | 78.000 |
| 205 | AC7188 | 384.800 | 0.260 | 0.418 | 0.220 | 1.256 | 52.000 |
| 206 | AC 7135 | 366.240 | 0.288 | 0.425 | 0.200 | 1.090 | 48.000 |
| 207 | AC 7147 | 321.200 | 0.120 | 0.432 | 0.160 | 1.124 | 40.000 |
| 208 | Polina dhan 2 | 79.680 | 0.054 | 0.190 | 0.220 | 0.819 | 12.000 |
| 209 | Gerwa thor | 278.400 | 0.090 | 0.697 | 0.590 | 1.204 | 30.000 |
| 210 | *Uttarbanga local-9 | 68.760 | 0.054 | 0.213 | 0.160 | 1.524 | 12.000 |
| 211 | Rohidhan-1 | 166.680 | 0.081 | 0.177 | 0.140 | 0.591 | 18.000 |
| 212 | *Joha | 116.380 | 0.110 | 0.240 | 0.208 | 0.917 | 22.000 |
| 213 | Manahar rathori | 84.700 | 0.070 | 0.323 | 0.090 | 1.023 | 14.000 |
| 214 | Ganrohibuna | 28.800 | 0.020 | 0.143 | 0.220 | 0.870 | 4.000 |
| 215 | Kadamful | 44.160 | 0.030 | 0.703 | 0.170 | 1.022 | 6.000 |
| 216 | *Jhingesal | 382.200 | 0.210 | 0.630 | 0.380 | 1.096 | 35.000 |
| 217 | Bakul dhan | 295.200 | 0.096 | 0.477 | 0.310 | 1.584 | 48.000 |
| 218 | *Gochi | 171.860 | 0.117 | 0.250 | 0.090 | 0.695 | 26.000 |
| 219 | Kaikee | 261.600 | 0.300 | 0.413 | 0.250 | 0.896 | 40.000 |
| 220 | *Palina dhan-1 | 104.352 | 0.048 | 0.273 | 0.300 | 1.032 | 16.000 |
| 221 | Bathi dhan | 258.160 | 0.105 | 0.235 | 0.250 | 0.790 | 35.000 |
| 222 | Kauka | 233.920 | 0.160 | 0.185 | 0.145 | 0.799 | 40.000 |
| 223 | Param nada | 321.615 | 0.090 | 0.214 | 0.260 | 0.996 | 45.000 |
| 224 | Sitaluchi | 367.125 | 0.275 | 0.165 | 0.180 | 0.809 | 55.000 |
| 225 | *Shayam | 694.400 | 0.130 | 0.830 | 0.260 | 1.309 | 80.000 |
| 226 | Kukurjali | 253.540 | 0.140 | 0.236 | 0.160 | 0.758 | 35.000 |
| 227 | Durudhan | 245.120 | 0.120 | 0.354 | 0.220 | 0.721 | 40.000 |
| 228 | Khara | 199.800 | 0.050 | 0.285 | 0.320 | 0.762 | 25.000 |
| 229 | Hetomari | 225.270 | 0.120 | 0.312 | 0.250 | 1.305 | 30.000 |
| 230 | *Dad ghani | 371.280 | 0.143 | 0.134 | 0.180 | 0.803 | 52.000 |
| 231 | Khajurchar | 326.320 | 0.120 | 0.242 | 0.170 | 0.786 | 40.000 |
| 232 | Pawan dhan | 487.740 | 0.220 | 0.253 | 0.230 | 0.701 | 55.000 |
| 233 | Meghjawain | 481.780 | 0.130 | 0.358 | 0.165 | 0.569 | 65.000 |
| 234 | *Basumati-B | 495.116 | 0.215 | 0.296 | 0.294 | 0.608 | 82.000 |
| 235 | Bharati | 251.600 | 0.200 | 0.264 | 0.210 | 0.720 | 40.000 |
| 236 | *Chatui muchi | 590.100 | 0.326 | 0.656 | 0.200 | 1.142 | 84.000 |
| 237 | Seshphal | 247.345 | 0.105 | 0.349 | 0.150 | 0.834 | 35.000 |
| 238 | Kaalibank | 234.330 | 0.120 | 0.284 | 0.230 | 0.831 | 30.000 |
| 239 | kaloghande swari | 191.040 | 0.120 | 0.412 | 0.180 | 0.511 | 30.000 |
| 240 | Jaldhapa | 323.480 | 0.120 | 0.385 | 0.200 | 0.546 | 40.000 |
| 241 | *Jhagri kartik | 210.540 | 0.061 | 0.948 | 0.512 | 1.945 | 22.000 |
| 242 | Chingrifuli | 388.962 | 0.210 | 0.248 | 0.280 | 0.507 | 42.000 |
| 243 | Lalkadhan | 266.000 | 0.140 | 0.251 | 0.220 | 0.478 | 35.000 |
| 244 | *Bharati | 149.712 | 0.080 | 0.124 | 0.200 | 0.825 | 16.000 |
| 245 | Moriadhan | 418.275 | 0.275 | 0.341 | 0.280 | 0.323 | 55.000 |
| 246 | Kashiyabinni | 226.350 | 0.270 | 0.335 | 0.160 | 0.406 | 45.000 |
| 247 | Kotki | 233.200 | 0.200 | 0.287 | 0.200 | 0.275 | 40.000 |
| 248 | *Sugandha-2 | 577.920 | 0.430 | 0.448 | 0.238 | 1.457 | 86.000 |
| 249 | Domnadhan | 284.924 | 0.152 | 0.291 | 0.180 | 0.458 | 38.000 |
| 250 | Kauka | 248.580 | 0.150 | 0.265 | 0.220 | 0.612 | 30.000 |
| 251 | AC44603 | 1490.000 | 1.120 | 1.250 | 0.500 | 1.678 | 100.000 |
| 252 | AC44585 | 1396.667 | 2.190 | 1.083 | 0.800 | 0.906 | 100.000 |
| 253 | AC44598 | 835.111 | 0.608 | 1.300 | 0.450 | 1.332 | 66.667 |
| 254 | AC44592 | 1900.000 | 1.630 | 1.133 | 0.400 | 1.286 | 100.000 |
| 255 | AC44646 | 1621.444 | 1.709 | 1.567 | 0.700 | 1.164 | 96.667 |
| 256 | AC44604 | 1526.667 | 2.720 | 0.150 | 0.400 | 1.399 | 100.000 |
| 257 | AC44597 | 1890.333 | 3.043 | 1.200 | 0.350 | 0.985 | 96.667 |
| 258 | AC44638 | 1536.000 | 0.875 | 2.583 | 0.500 | 1.231 | 93.333 |
| 259 | AC44595 | 1664.444 | 1.699 | 0.283 | 0.700 | 0.711 | 93.333 |
| 260 | AC44588 | 1420.000 | 1.940 | 0.250 | 0.500 | 0.841 | 100.000 |
| 261 | AC44591 | 1443.333 | 1.050 | 0.333 | 0.500 | 0.904 | 100.000 |
| 262 | AC44594 | 2086.667 | 2.430 | 1.350 | 0.700 | 0.789 | 100.000 |
| 263 | AC43737 | 1498.333 | 0.920 | 4.833 | 0.600 | 0.854 | 70.000 |
| 264 | AC43660 | 1215.500 | 0.625 | 4.367 | 0.400 | 0.727 | 45.000 |
| 265 | AC43732 | 1163.500 | 1.050 | 2.550 | 0.200 | 0.838 | 80.000 |
| 266 | AC43661 | 737.833 | 0.443 | 2.583 | 0.100 | 0.925 | 45.000 |
| 267 | AC43738 | 668.333 | 0.740 | 2.200 | 0.400 | 1.165 | 50.000 |
| 268 | AC43669 | 1809.667 | 0.950 | 4.633 | 0.350 | 0.982 | 70.000 |
| 269 | AC43663 | 1041.333 | 0.890 | 1.200 | 0.400 | 0.587 | 70.000 |
| 270 | AC43658 | 758.000 | 0.590 | 3.000 | 0.800 | 0.584 | 50.000 |
| 271 | AC43662 | 1287.833 | 0.985 | 2.633 | 0.700 | 0.754 | 80.000 |
| 272 | AC43670 | 1117.407 | 0.567 | 2.583 | 0.650 | 1.130 | 55.556 |
| 273 | AC43675 | 653.333 | 0.480 | 2.383 | 0.200 | 0.759 | 50.000 |
| 274 | AC43676 | 1131.167 | 0.945 | 2.183 | 0.200 | 0.567 | 65.000 |
|  | CV | 13.69 | 14.72 | 13.45 | 11.72 | 12.31 | 10.26 |
|  | LSD_5%_ | 174.913 | 0.153 | 0.892 | 0.148 | 0.318 | 10.261 |
