# Supplementary material for: Unraveling the genomic regions controlling the seed vigour index, root growth parameters and germination per cent in rice
Source: PLoS One. 2022 Jul 26;17(7):e0267303. doi: 10.1371/journal.pone.0267303 (PMC9321372; doi:10.1371/journal.pone.0267303)
Supplement: S2 Table — (DOCX) [file pone.0267303.s004.docx]

**Supplementary Table 2**. Markers information of the selected 136 SSR markers used for genotyping of 120 rice landraces

| **SL No** | **PR Name** | **Ch No** | **Position (Mb)** | **Forward** | **Reverse** | **Repeat**  **motif** | **AT** |
| --- | --- | --- | --- | --- | --- | --- | --- |
| 1 | **RM5310** | 1 | 41197253 | TAGACAAAGCAACGGGTTCC | CGGAAGCAGGAGAATCGTAG | (TC)12 | 55 |
| 2 | **RM582** | 1 | 9190478 | TCTGTTGCCGATTTGTTCG | AAATGGCTTACCTGCTGTCTC | (TC)20 | 55 |
| 3 | **RM13335** | 2 | 19213952 | TATGCCAAGAGGAATCCTGAAGC | GCACTCACACTGATCTGGACAGG | (GT)10 | 55 |
| 4 | **RM6275** | 2 | 7273421 | CACTGAGCCCTTTTGTCCTC | TCCCAGATCAGAATCGAAGG | (CTG)8 | 50 |
| 5 | **RM50** | 6 | 6300000 | ACTGTACCGGTCGAAGACG | AAATTCCACGTCAGCCTCC | (CTAT)4(CT)15 | 55 |
| 6 | **RM85** | 3 | 37200000 | CCAAAGATGAAACCTGGATTG | GCACAAGGTGAGCAGTCC | (TGG)5(TCT)12 | 55 |
| 7 | **RM222** | 10 | 2600000 | CTTAAATGGGCCACATGCG | CAAAGCTTCCGGCCAAAAG | (CT)18 | 55 |
| 8 | **RM247** | 12 | 3185384 | TAGTGCCGATCGATGTAACG | CATATGGTTTTGACAAAGCG | (CT)16 | 55 |
| 9 | **RM328** | 9 | 3,726,000 | CATAGTGGAGTATGCAGCTGC | CCTTCTCCCAGTCGTATCTG | (CAT)5 | 55 |
| 10 | **RM337** | 8 | 152299 | GTAGGAAAGGAAGGGCAGAG | CGATAGATAGCTAGATGTGGCC | (CTT)4-19-(CTT)8 | 55 |
| 11 | **RM340** | 6 | 28599181 | GGTAAATGGACAATCCTATGGC | GACAAATATAAGGGCAGTGTGC | (CTT)8T3(CTT)14 | 55 |
| 12 | **RM470** | 4 | 28,090,431 | TCCTCATCGGCTTCTTCTTC | AGAACCCGTTCTACGTCACG | (CTT)14 | 55 |
| 13 | **RM472** | 1 | 37889084 | CCATGGCCTGAGAGAGAGAG | AGCTAAATGGCCATACGGTG | (GA)21 | 55 |
| 14 | **RM506** | 8 | 435648-35677 | CGAGCTAACTTCCGTTCTGG | GCTACTTGGGTAGCTGACCG | (CT)13 | 55 |
| 15 | **RM1812** | 11 | 2405106 | CAGCTAGTGAGCTCCTAGTG | GCTAACCCACCAACTTATTC | (AT)16 | 55 |
| 16 | **RM3701** | 11 | 8100974 | GAGCTAGAGGGAGGAGGTGC | TTGACTGATAGCCGATTGGG | (GA)15 | 55 |
| 17 | **RM6947** | 12 | 23974120 | ATTAAACGTCCACTGCTGGC | GCTAGGTTAGTGGTGCAGGG | (TTC)8 | 55 |
| 18 | **RM14978** | 3 | 13880447 | TATCTGCAGGTGCGTGTAAATGG | GCATATAGAGCGAGTAAGCGAGAGG | (TC)10 | 55 |
| 19 | **RM18776** | 5 | 21661718 | CTCCAGGAGGGTACAAATTCTGC | CCATTGGAACATAGCAAGTGATCG | (GA)13 | 53 |
| 20 | **RM22034** | 7 | 26403619 | CCAGTTTATCTTCTGCACCTTCTCG | TCTTTGAGCAGATGGCTAACAAGG | (ATTA)5 | 53 |
| 21 | **RM24161** | 9 | 12311985 | GTATGGCGAGACCCTACAGACC | GACCCACTTAATGTGTCACAAGG | (TATT)5 | 54 |
| 22 | **RM223** | 8 | 20650060 | GAGTGAGCTTGGGCTGAAAC | GAAGGCAAGTCTTGGCACTG | (CT)25 | 55 |
| 23 | **RM440** | 5 | 19912517 | CATGCAACAACGTCACCTTC | ATGGTTGGTAGGCACCAAAG | (CTT)22 | 55 |
| 24 | **RM201** | 9 | 20174289 | CTCGTTTATTACCTACAGTACC | CTACCTCCTTTCTAGACCGATA | (CT)17 | 55 |
| 25 | **RM216** | 10 | 5,352,766 | GCATGGCCGATGGTAAAG | TGTATAAAACCACACGGCCA | (CT)18 | 55 |
| 26 | **RM258** | 10 | 18014265 | TGCTGTATGTAGCTCGCACC | TGGCCTTTAAAGCTGTCGC | (GA)21(GGA)3 | 55 |
| 27 | **RM286** | 11 | 383711 | GGCTTCATCTTTGGCGAC | CCGGATTCACGAGATAAACTC | (GA)16 | 55 |
| 28 | **RM3735** | 4 | 26210755 | GCGACCGATCAGCTAGCTAG | ATAACTCCTCCCTTGCTGCC | (GA)16 | 55 |
| 29 | **RM1347** | 2 | 5314190 | AACAAATTAAACTGCCAAG | GTCTTATCATCAGAACTGGA | (AG)23 | 55 |
| 30 | **RM7571** | 7 | 10369821 | CCTTATGCCCCCTTCCTTAC | TCGTCTCATGGAGCCACC | (TCTA)6 | 61 |
| 31 | **RM14723** | 3 | 9,223,269 | GCAAAGTCCTTTGGACAGGTAGC | CGTCCCAGATCAAAGTACACTCTTCC | (GA)29 | 54 |
| 32 | **RM103** | 6 | 30889151 | CTTCCAATTCAGGCCGGCTGGC | CGCCACAGCTGACCATGCATGC | (GAA)5 | 55 |
| 33 | **RM315** | 1 | 36734135 | GAGGTACTTCCTCCGTTTCAC | AGTCAGCTCACTGTGCAGTG | (AT)4(GT)10 | 55 |
| 34 | **RM225** | 6 | 3416533 | TGCCCATATGGTCTGGATG | GAAAGTGGATCAGGAAGGC | (CT)18 | 55 |
| 35 | **RM486** | 1 | 34955554 | CCCCCCTCTCTCTCTCTCTC | TAGCCACATCAACAGCTTGC | (CT)14 | 55 |
| 36 | **RM256** | 8 | 24270635 | GACAGGGAGTGATTGAAGGC | GTTGATTTCGCCAAGGGC | (CT)21 | 55 |
| 37 | **RM1113** | 4 | 34085697 | GGGCGCATGTGTATTTCTTC | TGGGGAAAAACCACAAGCC | (AG)12 | 55 |
| 38 | **RM3423** | 4 | 30,957,747 | AGCAGGCATATAAAGGTGCC | TGGCCTCAGATTCAGGAAAC | (CT)18 | 50 |
| 39 | **RM6100** | 10 | 18,816,637 | TCCTCTACCAGTACCGCACC | GCTGGATCACAGATCATTGC | (CGA)8 | 50 |
| 40 | **RM590** | 10 | 23043156 | CATCTCCGCTCTCCATGC | GGAGTTGGGGTCTTGTTCG | (TCT)10 | 55 |
| 41 | **RM5793** | 7 | 17489638 | ACTCTCTTGCGCAACTCCTC | GATAATGCTAGCTGCTGGCC |  | 50 |
| 42 | **RM405** | 5 | 3073406 | TCACACACTGACAGTCTGAC | AATGTGGCACGTGAGGTAAG | (AC)14 | 55 |
| 43 | **RM547** | 8 | 5591403 | TAGGTTGGCAGACCTTTTCG | GTCAAGATCATCCTCGTAGCG | (ATT)19 | 55 |
| 44 | **RM7364** | 9 | 9561213 | TTCGTGGATGGAGGGAGTAC | AGCGTTTGTAGGAGTGCCAC | (CTAT)9 | 50 |
| 45 | **RM205** | 9 | 22720624 | CTGGTTCTGTATGGGAGCAG | CTGGCCCTTCACGTTTCAGTG | (CT)25 | 55 |
| 46 | **RM167** | 11 | 4073024 | GATCCAGCGTGAGGAACACGT | AGTCCGACCACAAGGTGCGTTGTC | (GA)16 | 53 |
| 47 | **RM229** | 11 | 18,407,879 | CACTCACACGAACGACTGAC | CGCAGGTTCTTGTGAAATGT | (TC)11(CT)5C3(CT)5 | 50 |
| 48 | **RM20A** | 12 | 970538 | ATCTTGTCCCTGCAGGTCAT | GAAACAGAGGCACATTTCATTG | (ATT)14 | 55 |
| 49 | **RM235** | 12 | 26107904 | AGAAGCTAGGGCTAACGAAC | TCACCTGGTCAGCCTCTTTC | (CT)24 | 55 |
| 50 | **RM7003** | 12 | 6775083 | GGCAGACATACAGCTTATAGGC | TGCAAATGAACCCCTCTAGC | (AAAC)6 | 50 |
| 51 | **RM5436** | 7 | 9074712 | CAAAGGGGGTGTCCTCTATG | GTTGCTCGTCCTACATGTGC |  | 50 |
| 52 | **RM25181** | 10 | 8849270 | AAAGAGCTTCCCTAATGGCTTCG | GAGAGAATGACCTCTCCCAAGACC | (TTC)22 | 55 |
| 53 | **RM469** | 6 | 564,135 | AGCTGAACAAGCCCTGAAAG | GACTTGGGCAGTGTGACATG | (AG)15 | 55 |
| 54 | **RM6547** | 1 | 34693224 | TCCATCCTTCTCCTCTCGTG | AGCCACCCCCATATATAGCC | (GCT)9 | 50 |
| 55 | **RM152** | 8 | 682963 | GAAACCACCACACCTCACCG | CCGTAGACCTTCTTGAAGTAG | (GGC)10 | 55 |
| 56 | **RM148** | 3 | 35835805 | ATACAACATTAGGGATGAGGCTGG | TCCTTAAAGGTGGTGCAATGCGAG | (TG)12 | 50 |
| 57 | **RM421** | 5 | 23976333 | AGCTCAGGTGAAACATCCAC | ATCCAGAATCCATTGACCCC | (AGAT)6 | 55 |
| 58 | **RM2634** | 2 | 20495111 | GATTGAAAATTAGAGTTTGCAC | TGCCGAGATTTAGTCAACTA | (AT)31 | 55 |
| 59 | **RM248** | 7 | 29,339,845 | TCCTTGTGAAATCTGGTCCC | GTAGCCTAGCATGGTGCATG | (CT)25 | 55 |
| 60 | **RM7179** | 6 | 19728535 | CACGTGTCAGCTTAAGAGCG | TTACATCATAAGCCCGCAGG | (ATAG)6 | 50 |
| 61 | **RM215** | 9 | 21189110 | CAAAATGGAGCAGCAAGAGC | TGAGCACCTCCTTCTCTGTAG | (CT)16 | 55 |
| 62 | **RM324** | 2 | 11389704 | CTGATTCCACACACTTGTGC | GATTCCACGTCAGGATCTTC | (CAT)21 | 55 |
| 63 | **RM317** | 4 | 29060978 | CATACTTACCAGTTCACCGCC | CTGGAGAGTGTCAGCTAGTTGA | (GC)4(GT)18 | 55 |
| 64 | **RM174** | 2 | 7006085 | AGCGACGCCAAGACAAGTCGGG | TCCACGTCGATCGACACGACGG | (AGG)7(GA)10 | 67 |
| 65 | **RM556** | 8 | 22339816 | ACTCCAAACCTCACTGCACC | TAGCACACTGAACAGCTGGC | (CCAG)6 | 55 |
| 66 | **RM257** | 9 | 17719660 | CAGTTCCGAGCAAGAGTACTC | GGATCGGACGTGGCATATG | (CT)24 | 55 |
| 67 | **RM502** | 8 | 26492117 | GCGATCGATGGCTACGAC | ACAACCCAACAAGAAGGACG | (TG)10 | 55 |
| 68 | **RM331** | 8 | 12294124 | GAACCAGAGGACAAAAATGC | CATCATACATTTGCAGCCAG | [(CTT)4GTT]2(CTT)11 | 55 |
| 69 | **RM403** | 1 | 29384585 | GCTGTGCATGCAAGTTCATG | ATGGTCCTCATGTTCATGGC | (GA)8 | 55 |
| 70 | **RM309** | 12 | 21454591 | GTAGATCACGCACCTTTCTGG | AGAAGGCCTCCGGTGAAG | (GT)13 | 55 |
| 71 | **RM6641** | 2 | 4633966 | GGGTCTCGATTCTCAGTTGG | CAGAACCACTCATGCACACC | (GTA)14 | 55 |
| 72 | **RM3** | 6 | 19499320 | ACACTGTAGCGGCCACTG | CCTCCACTGCTCCACATCTT | (GA)2GG(GA)25 | 55 |
| 73 | **RM594** | 1 | 15158295 | GCCACCAGTAAAAGCAATAC | TTGATCTGCTAGTGAGACCC | (GA)n | 55 |
| 74 | **RM3392** | 3 | 3825907 | GTCCAATGATTCGTTCCCAC | CTTCACCGTTCACCAATTCC | (CT)17 | 55 |
| 75 | **RM1278** | 3 | 4561347 | ATATAAAGGTGGCACGACAG | GCACTTGAACTCTAATTCTCC | (AG)17 | 55 |
| 76 | **RM168** | 3 | 28091534 | TGCTGCTTGCCTGCTTCCTTT | GAAACGAATCAATCCACGGC | T15(GT)14 | 50 |
| 77 | **RM3375** | 1 | 18729953 | TTGACCTCCTCCTCCACAAC | TTGCAAGGAAACTAGGAGGG | (CT)16 | 55 |
| 78 | **RM282** | 3 | 12407382 | CTGTGTCGAAAGGCTGCAC | CAGTCCTGTGTTGCAGCAAG | (GA)15 | 55 |
| 79 | **RM26632** | 11 | 14702841 | CCAATCACAACCCTCCATCACC | CCAAAGAGCAACATTGGTTGTGC | (TCTT)9 | 50 |
| 80 | **RM1341** | 11 | 19677083 | AACCTGGAGGTGCTGGTCTC | TTTCTCCCCCCCAACCAC |  | 50 |
| 81 | **RM4112** | 11 | 24646850 | TGGCAAAGTCAGTAGTCCTTCCACAA | GCCATTCCCCCAACAGCTCC | (TA)14 | 55 |
| 82 | **RM20377** | 6 | 24,320,992 | GTGTGTGATGTGCATGTTTCTGC | CATGTGATGCCCTGTAGGAACC | (CT)33 | 50 |
| 83 | **RM210** | 8 | 22471837 | TCACATTCGGTGGCATTG | CGAGGATGGTTGTTCACTTG | (CT)23 | 55 |
| 84 | **RM218** | 3 | 8405368 | TGGTCAAACCAAGGTCCTTC | GACATACATTCTACCCCCGG | (TC)24ACT5(GT)11 | 55 |
| 85 | **RM494** | 6 | 31088146 | GGGAGGGGATCGAGATAGAC | TTTAACCTTCCTTCCGCTCC | (AGA)16 | 55 |
| 86 | **RM336** | 7 | 21,871,205 | CTTACAGAGAAACGGCATCG | GCTGGTTTGTTTCAGGTTCG | (CTT)18 | 55 |
| 87 | **RM3475** | 1 | 26041024 | GTCGGTTTGCCTAGTTGAGC | TTCCTCGGTGTATGGGTCTC | (CT)22 | 55 |
| 88 | **RM480** | 5 | 27,313,250 | GCTCAAGCATTCTGCAGTTG | GCGCTTCTGCTTATTGGAAG | (AC)30 | 55 |
| 89 | **RM566** | 8 | 14704764 | ACCCAACTACGATCAGCTCG | CTCCAGGAACACGCTCTTTC | (CCAG)6 | 55 |
| 90 | **RM11701** | 1 | 32026621 | CTGGTGGAGTTGCAGTGCCTCTAGC | CCTTGCTGCTTTCTCATTGAAACTGG | (CT)18 | 56 |
| 91 | **RM220** | 1 | 4424392 | GGAAGGTAACTGTTTCCAAC | GAAATGCTTCCCACATGTCT | (CT)17 | 55 |
| 92 | **RM488** | 1 | 24807508 | CAGCTAGGGTTTTGAGGCTG | TAGCAACAACCAGCGTATGC | (GA)17 | 55 |
| 93 | **RM6374** | 2 | 15181966 | TGAGGACGCTGATTGTCAAC | GCTGCCCCTATTATTTCACC | (GAA)16 | 55 |
| 94 | **RM233** | 2 | 2069848 | CCAAATGAACCTACATGTTG | GCATTGCAGACAGCTATTGA | (CT)20 | 55 |
| 95 | **RM112** | 2 | 32013785 | GGGAGGAGAGGCAAGCGGAGAG | AGCCGGTGCAGTGGACGGTGAC | (GAA)5 | 55 |
| 96 | **RM13600** | 2 | 24246249 | GGTTAACCTTTCTCGCTCTTTGG | ATGATCCAAACCCACTGTCTTCC | (AG)11 | 50 |
| 97 | **RM495** | 1 | 215956 | AATCCAAGGTGCAGAGATGG | CAACGATGACGAACACAACC | (CTG)7 | 55 |
| 98 | **RM493** | 1 | 12280117 | TAGCTCCAACAGGATCGACC | GTACGTAAACGCGGAAGGTG | (CTT)9 | 55 |
| 99 | **RM444** | 9 | 5925016 | GCTCCACCTGCTTAAGCATC | TGAAGACCATGTTCTGCAGG | (AT)12 | 55 |
| 100 | **RM468** | 3 | 32674852 | CCCTTCCTTGTTGTGGCTAC | TGATTTCTGAGAGCCAACCC | (TAT)8 | 55 |
| 101 | **RM6054** | 5 | 22779263 | CCCTCCGTACGGATACACAC | CTCTTCGGCTTCATCTCCTC | (CCG)12 | 55 |
| 102 | **RM509** | 5 | 16324561 | TAGTGAGGGAGTGGAAACGG | ATCGTCCCCACAATCTCATC | (TC)11 | 55 |
| 103 | **RM5638** | 1 | 20934810 | GGCTTCCTCATCGCCATC | CTGAGCAGCATTCCAGTCTG | (AAG)13 | 55 |
| 104 | **RM8044** | 7 | 24,195,172 | AGTACTTGTCTCCTTAGCAG | CAATATTCACTCAACTCTCA | (CTT)18 | 55 |
| 105 | **RM8271** | 8 | 7616956-617315 | TCTTGAGAAATCTGCCATTC | ACTGATGTGCATTTCGTC | (AG)32 | 55 |
| 106 | **RM171** | 10 | 19048795 | AACGCGAGGACACGTACTTAC | ACGAGATACGTACGCCTTTG | (GATG)5 | 55 |
| 107 | **RM16686** | 4 | 14718643 | GGCACTGCTTGCATATGGATCG | TGCCGGCGAACTTATCCTCTCC | (GGA)10 | 53 |
| 108 | **RM434** | 9 | 15662573 | GCCTCATCCCTCTAACCCTC | CAAGAAAGATCAGTGCGTGG | (TC)12 | 55 |
| 109 | **RM6091** | 11 | 13405326 | GCTGTCCTGTCCTTGAATCC | TGGTAGGCTGGTGACATGC | (CCT)11 | 50 |
| 110 | **RM209** | 11 | 17808335 | ATATGAGTTGCTGTCGTGCG | CAACTTGCATCCTCCCCTCC | (CT)18 | 55 |
| 111 | **RM245** | 9 | 22300000 | ATGCCGCCAGTGAATAGC | CTGAGAATCCAATTATCTGGGG | (CT)14 | 55 |
| 112 | **RM1089** | 5 | 5356127 | CAGAAGGATTATCTCGATACC | AATAGGGCTTGAAATAAATTG | (AC)33 | 55 |
| 113 | **RM228** | 10 | 22243157 | CTGGCCATTAGTCCTTGG | GCTTGCGGCTCTGCTTAC | (CA)6(GA)36 | 55 |
| 114 | **RM401** | 4 | 13154172 | TGGAACAGATAGGGTGTAAGGG | CCGTTCACAACACTATACAAGC | (CT)15 | 55 |
| 115 | **RM11** | 7 | 19256914 | TCTCCTCTTCCCCCGATC | ATAGCGGGCGAGGCTTAG | (GA)17 | 55 |
| 116 | **RM3351** | 5 | 20696671 | ATGGAAGGAATGGAGGTGAG | TACCCCTACGTCGATCGATC | (CT)15 | 55 |
| 117 | **RM5749** | 4 | 19950587 | GTGACCACATCTATATCGCTCG | ATGGCAAGGTTGGATCAGTC | (ACT)8 | 55 |
| 118 | **RM335** | 4 | 688353 | GTACACACCCACATCGAGAAG | GCTCTATGCGAGTATCCATGG | (CTT)25 | 55 |
| 119 | **RM144** | 11 | 28281693 | TGCCCTGGCGCAAATTTGATCC | GCTAGAGGAGATCAGATGGTAGTGCATG | (ATT)11 | 55 |
| 120 | **RM300** | 2 | 13191380 | GCTTAAGGACTTCTGCGAACC | CAACAGCGATCCACATCATC | (GTT)14 | 55 |
| 121 | **RM1132** | 7 | 23984489 | ATCACCTGAGAAACATCCGG | CTCCTCCCACGTCAAGGTC | (AG)12 | 55 |
| 122 | **RM400** | 6 | 28431560 | ACACCAGGCTACCCAAACTC | CGGAGAGATCTGACATGTGG | (ATA)63 | 55 |
| 123 | **RM471** | 4 | 18824746 | ACGCACAAGCAGATGATGAG | GGGAGAAGACGAATGTTTGC | (GA)12 | 55 |
| 124 | **RM243** | 1 | 7970722 | GATCTGCAGACTGCAGTTGC | AGCTGCAACGATGTTGTCC | (CT)18 | 55 |
| 125 | **RM467** | 10 | 13488471 | GGTCTCTCTCTCTCTCTCTCTCTC | CTCCTGACAATTCAACTGCG | (TC)21 | 55 |
| 126 | **RM564** | 3 | 18587434 | CATGGCCTTGTGTATGCATC | ATGCAGAGGATTGGCTTGAG | (GT)14 | 55 |
| 127 | **RM8007** | 7 | 7710329 | AATAGGATGGATCATGGATA | CATCTCATCAGGAACCTAAC | (AT)40 | 55 |
| 128 | **RM441** | 11 | 6081100 | ACACCAGAGAGAGAGAGAGAGAG | TCTGCAACGGCTGATAGATG | (AG)13 | 55 |
| 129 | **RM518** | 4 | 2030135 | CTCTTCACTCACTCACCATGG | ATCCATCTGGAGCAAGCAAC | (TC)15 | 55 |
| 130 | **RM253** | 6 | 5425408 | TCCTTCAAGAGTGCAAAACC | GCATTGTCATGTCGAAGCC | (GA)25 | 55 |
| 131 | **RM274** | 5 | 26848154 | CCTCGCTTATGAGAGCTTCG | CTTCTCCATCACTCCCATGG | (GA)15-7-(CGG)5 | 55 |
| 132 | **RM242** | 9 | 18810067 | GGCCAACGTGTGTATGTCTC | TATATGCCAAGACGGATGGG | (CT)26 | 55 |
| 133 | **RM3231** | 8 | 3838134 | AACACGAAGACCGGCCTC | CAGGTAGGAGCATGAGAGCC | (CT)12 | 55 |
| 134 | **RM5687** | 4 | 15742285 | GATCGCTGGCGATTGATC | GACTTGTGGGGTGGTTTTTG | (AAT)17 | 50 |
| 135 | **RM5626** | 3 | 24864350 | GCAGACGAGATGAGATCG | GTAGAGGATGGGCAGCAG | (AAG)11 | 55 |
| 136 | **RM452** | 2 | 9563257 | CTGATCGAGAGCGTTAAGGG | GGGATCAAACCACGTTTCTG | (GTC)9 | 55 |
