# Supplementary material for: Unraveling the genomic regions controlling the seed vigour index, root growth parameters and germination per cent in rice
Source: PLoS One. 2022 Jul 26;17(7):e0267303. doi: 10.1371/journal.pone.0267303 (PMC9321372; doi:10.1371/journal.pone.0267303)
Supplement: S3 Table — (DOCX) [file pone.0267303.s005.docx]

**Supplementary Table 3.** Estimation of genetic diversity parameters using 136 SSR markers loci in a panel population containing 120 rice landraces.

| Sl. No. | Marker | No. of Alleles | Range of amplicon (bp) | Major allele frquency | Gene diversity | Heterozygosity | PIC | Inbreeding coefficient  (f) |
| --- | --- | --- | --- | --- | --- | --- | --- | --- |
| 1 | RM5310 | 4 | 140-190 | 0.783 | 0.3671 | 0.033 | 0.343 | 0.910 |
| 2 | RM582 | 4 | 210-245 | 0.708 | 0.4657 | 0.033 | 0.433 | 0.929 |
| 3 | RM13335 | 4 | 160-180 | 0.563 | 0.5316 | 0.008 | 0.435 | 0.984 |
| 4 | RM6275 | 4 | 140-160 | 0.721 | 0.4466 | 0.058 | 0.411 | 0.870 |
| 5 | RM50 | 4 | 190-205 | 0.400 | 0.6886 | 0.025 | 0.630 | 0.964 |
| 6 | RM85 | 4 | 80-110 | 0.413 | 0.6753 | 0.125 | 0.615 | 0.816 |
| 7 | RM222 | 4 | 210-250 | 0.629 | 0.5573 | 0.025 | 0.519 | 0.956 |
| 8 | RM247 | 5 | 140-200 | 0.500 | 0.5970 | 0.067 | 0.519 | 0.889 |
| 9 | RM328 | 3 | 185-200 | 0.567 | 0.5800 | 0.000 | 0.513 | 1.000 |
| 10 | RM337 | 6 | 155-400 | 0.446 | 0.6684 | 0.117 | 0.612 | 0.827 |
| 11 | RM340 | 5 | 100-220 | 0.713 | 0.4539 | 0.100 | 0.415 | 0.781 |
| 12 | RM470 | 5 | 60-140 | 0.463 | 0.6904 | 0.833 | 0.644 | -0.203 |
| 13 | RM472 | 3 | 290-410 | 0.513 | 0.5077 | 0.092 | 0.387 | 0.821 |
| 14 | RM506 | 3 | 120-130 | 0.683 | 0.4594 | 0.133 | 0.390 | 0.712 |
| 15 | RM1812 | 3 | 130-140 | 0.442 | 0.6065 | 0.000 | 0.523 | 1.000 |
| 16 | RM3701 | 4 | 160-260 | 0.675 | 0.4840 | 0.492 | 0.428 | -0.012 |
| 17 | RM6947 | 3 | 150-160 | 0.883 | 0.2117 | 0.000 | 0.199 | 1.000 |
| 18 | RM14978 | 3 | 240-250 | 0.417 | 0.6394 | 0.000 | 0.563 | 1.000 |
| 19 | RM18776 | 3 | 175-200 | 0.846 | 0.2673 | 0.025 | 0.242 | 0.907 |
| 20 | RM22034 | 3 | 75-85 | 0.917 | 0.1550 | 0.000 | 0.147 | 1.000 |
| 21 | RM24161 | 4 | 270-290 | 0.542 | 0.6121 | 0.117 | 0.552 | 0.811 |
| 22 | RM223 | 5 | 110-170 | 0.654 | 0.5360 | 0.058 | 0.504 | 0.892 |
| 23 | RM440 | 5 | 160-210 | 0.408 | 0.6891 | 0.258 | 0.634 | 0.628 |
| 24 | RM201 | 3 | 150-160 | 0.479 | 0.5778 | 0.025 | 0.486 | 0.957 |
| 25 | RM216 | 4 | 145-160 | 0.513 | 0.6393 | 0.125 | 0.583 | 0.806 |
| 26 | RM258 | 3 | 140-150 | 0.383 | 0.6517 | 0.000 | 0.576 | 1.000 |
| 27 | RM286 | 4 | 100-130 | 0.471 | 0.6325 | 0.100 | 0.562 | 0.843 |
| 28 | RM3735 | 4 | 135-500 | 0.333 | 0.7252 | 0.958 | 0.674 | -0.318 |
| 29 | RM1347 | 3 | 100-110 | 0.517 | 0.5661 | 0.000 | 0.475 | 1.000 |
| 30 | RM7571 | 3 | 130-140 | 0.713 | 0.4334 | 0.008 | 0.373 | 0.981 |
| 31 | RM14723 | 4 | 220-250 | 0.492 | 0.6435 | 0.200 | 0.581 | 0.691 |
| 32 | RM103 | 3 | 255-330 | 0.492 | 0.5588 | 0.767 | 0.461 | -0.369 |
| 33 | RM315 | 3 | 135-140 | 0.867 | 0.2350 | 0.000 | 0.214 | 1.000 |
| 34 | RM225 | 3 | 135-150 | 0.525 | 0.5474 | 0.183 | 0.449 | 0.667 |
| 35 | RM486 | 3 | 130-140 | 0.654 | 0.4685 | 0.108 | 0.380 | 0.770 |
| 36 | RM256 | 3 | 110-150 | 0.721 | 0.4112 | 0.058 | 0.339 | 0.859 |
| 37 | RM1113 | 3 | 150-180 | 0.671 | 0.4568 | 0.058 | 0.373 | 0.873 |
| 38 | RM3423 | 3 | 125-140 | 0.500 | 0.5749 | 0.000 | 0.484 | 1.000 |
| 39 | RM6100 | 3 | 170-180 | 0.442 | 0.6432 | 0.033 | 0.569 | 0.949 |
| 40 | RM590 | 3 | 140-150 | 0.725 | 0.4307 | 0.067 | 0.384 | 0.846 |
| 41 | RM5793 | 3 | 115-130 | 0.633 | 0.5249 | 0.017 | 0.464 | 0.969 |
| 42 | RM405 | 3 | 100-110 | 0.675 | 0.4913 | 0.000 | 0.441 | 1.000 |
| 43 | RM547 | 5 | 190-300 | 0.471 | 0.5732 | 0.167 | 0.481 | 0.711 |
| 44 | RM7364 | 5 | 180-250 | 0.621 | 0.5734 | 0.167 | 0.541 | 0.711 |
| 45 | RM205 | 3 | 130-180 | 0.621 | 0.5320 | 0.025 | 0.467 | 0.953 |
| 46 | RM167 | 4 | 130-180 | 0.704 | 0.4628 | 0.100 | 0.421 | 0.786 |
| 47 | RM229 | 4 | 120-140 | 0.358 | 0.7071 | 0.133 | 0.652 | 0.813 |
| 48 | RM20A | 3 | 230-240 | 0.625 | 0.5332 | 0.017 | 0.472 | 0.969 |
| 49 | RM235 | 5 | 100-145 | 0.396 | 0.7185 | 0.175 | 0.671 | 0.758 |
| 50 | RM7003 | 4 | 100-110 | 0.667 | 0.5018 | 0.083 | 0.453 | 0.835 |
| 51 | RM5436 | 4 | 155-190 | 0.442 | 0.6210 | 0.058 | 0.545 | 0.907 |
| 52 | RM25181 | 5 | 130-160 | 0.379 | 0.7098 | 0.167 | 0.660 | 0.767 |
| 53 | RM469 | 3 | 100-110 | 0.621 | 0.5235 | 0.042 | 0.452 | 0.921 |
| 54 | RM6547 | 3 | 155-165 | 0.867 | 0.2399 | 0.017 | 0.226 | 0.931 |
| 55 | RM152 | 4 | 145-155 | 0.508 | 0.6282 | 0.017 | 0.565 | 0.974 |
| 56 | RM148 | 2 | 140-150 | 0.675 | 0.4388 | 0.083 | 0.342 | 0.812 |
| 57 | RM421 | 3 | 250-260 | 0.458 | 0.6307 | 0.000 | 0.555 | 1.000 |
| 58 | RM2634 | 3 | 100-120 | 0.379 | 0.6581 | 0.025 | 0.584 | 0.962 |
| 59 | RM248 | 4 | 75-115 | 0.346 | 0.7325 | 0.117 | 0.684 | 0.842 |
| 60 | RM7179 | 5 | 50-250 | 0.325 | 0.7654 | 0.358 | 0.727 | 0.535 |
| 61 | RM215 | 3 | 155-165 | 0.617 | 0.4907 | 0.017 | 0.392 | 0.966 |
| 62 | RM324 | 4 | 220-260 | 0.542 | 0.6353 | 0.158 | 0.590 | 0.753 |
| 63 | RM317 | 3 | 150-160 | 0.725 | 0.4032 | 0.000 | 0.328 | 1.000 |
| 64 | RM174 | 3 | 230-270 | 0.508 | 0.6207 | 0.067 | 0.551 | 0.893 |
| 65 | RM556 | 3 | 190-210 | 0.842 | 0.2788 | 0.033 | 0.260 | 0.881 |
| 66 | RM257 | 4 | 130-155 | 0.408 | 0.6632 | 0.233 | 0.595 | 0.651 |
| 67 | RM502 | 3 | 260-265 | 0.808 | 0.3182 | 0.000 | 0.281 | 1.000 |
| 68 | RM331 | 4 | 95-115 | 0.483 | 0.6639 | 0.058 | 0.611 | 0.913 |
| 69 | RM403 | 4 | 110-130 | 0.596 | 0.5702 | 0.083 | 0.515 | 0.855 |
| 70 | RM309 | 3 | 180-190 | 0.696 | 0.4601 | 0.025 | 0.405 | 0.946 |
| 71 | RM6641 | 3 | 140-145 | 0.567 | 0.5828 | 0.000 | 0.517 | 1.000 |
| 72 | RM3 | 3 | 110-120 | 0.383 | 0.6628 | 0.033 | 0.589 | 0.950 |
| 73 | RM594 | 3 | 300-320 | 0.588 | 0.5579 | 0.008 | 0.488 | 0.985 |
| 74 | RM3392 | 4 | 160-180 | 0.504 | 0.6152 | 0.108 | 0.545 | 0.825 |
| 75 | RM1278 | 3 | 135-150 | 0.783 | 0.3607 | 0.067 | 0.329 | 0.817 |
| 76 | RM168 | 3 | 95-125 | 0.625 | 0.5099 | 0.150 | 0.431 | 0.708 |
| 77 | RM3375 | 3 | 190-200 | 0.567 | 0.5761 | 0.033 | 0.506 | 0.943 |
| 78 | RM282 | 3 | 140-150 | 0.725 | 0.4363 | 0.000 | 0.395 | 1.000 |
| 79 | RM26632 | 4 | 450-550 | 0.363 | 0.7008 | 0.158 | 0.644 | 0.776 |
| 80 | RM1341 | 3 | 170-190 | 0.613 | 0.5289 | 0.025 | 0.455 | 0.953 |
| 81 | RM4112 | 3 | 160-170 | 0.488 | 0.6227 | 0.158 | 0.549 | 0.748 |
| 82 | RM20377 | 4 | 300-380 | 0.771 | 0.3695 | 0.067 | 0.326 | 0.821 |
| 83 | RM210 | 5 | 130-180 | 0.363 | 0.7341 | 0.700 | 0.687 | 0.051 |
| 84 | RM218 | 4 | 130-160 | 0.583 | 0.5849 | 0.033 | 0.531 | 0.943 |
| 85 | RM494 | 5 | 130-180 | 0.383 | 0.7168 | 0.025 | 0.670 | 0.965 |
| 86 | RM336 | 5 | 105-160 | 0.383 | 0.7107 | 0.092 | 0.661 | 0.872 |
| 87 | RM3475 | 4 | 135-160 | 0.450 | 0.6561 | 0.042 | 0.591 | 0.937 |
| 88 | RM480 | 4 | 190-210 | 0.538 | 0.6177 | 0.025 | 0.561 | 0.960 |
| 89 | RM566 | 4 | 150-200 | 0.433 | 0.6561 | 0.017 | 0.591 | 0.975 |
| 90 | RM11701 | 3 | 210-230 | 0.642 | 0.4713 | 0.000 | 0.375 | 1.000 |
| 91 | RM220 | 6 | 85-130 | 0.358 | 0.7450 | 0.183 | 0.703 | 0.756 |
| 92 | RM488 | 6 | 155-200 | 0.321 | 0.7496 | 0.192 | 0.708 | 0.746 |
| 93 | RM6374 | 6 | 130-160 | 0.338 | 0.7715 | 0.075 | 0.737 | 0.904 |
| 94 | RM233 | 5 | 130-160 | 0.350 | 0.7273 | 0.233 | 0.680 | 0.681 |
| 95 | RM112 | 3 | 130-135 | 0.875 | 0.2224 | 0.000 | 0.204 | 1.000 |
| 96 | RM13600 | 4 | 105-130 | 0.479 | 0.6623 | 0.100 | 0.607 | 0.850 |
| 97 | RM495 | 3 | 145-165 | 0.600 | 0.5599 | 0.033 | 0.499 | 0.941 |
| 98 | RM493 | 7 | 180-250 | 0.283 | 0.8126 | 0.558 | 0.787 | 0.317 |
| 99 | RM444 | 5 | 180-240 | 0.321 | 0.7727 | 0.158 | 0.737 | 0.797 |
| 100 | RM468 | 3 | 210-220 | 0.771 | 0.3789 | 0.025 | 0.346 | 0.935 |
| 101 | RM6054 | 3 | 120-130 | 0.925 | 0.1415 | 0.017 | 0.137 | 0.883 |
| 102 | RM509 | 3 | 165-170 | 0.758 | 0.3949 | 0.000 | 0.360 | 1.000 |
| 103 | RM5638 | 6 | 190-240 | 0.613 | 0.5870 | 0.133 | 0.558 | 0.775 |
| 104 | RM8044 | 6 | 240-300 | 0.279 | 0.7608 | 0.233 | 0.721 | 0.695 |
| 105 | RM8271 | 5 | 180-250 | 0.404 | 0.7233 | 0.133 | 0.679 | 0.817 |
| 106 | RM171 | 4 | 380-420 | 0.517 | 0.6333 | 0.058 | 0.575 | 0.909 |
| 107 | RM16686 | 3 | 90-100 | 0.417 | 0.6550 | 0.000 | 0.581 | 1.000 |
| 108 | RM434 | 4 | 250-280 | 0.567 | 0.5948 | 0.025 | 0.537 | 0.958 |
| 109 | RM6091 | 4 | 70-80 | 0.817 | 0.3185 | 0.000 | 0.299 | 1.000 |
| 110 | RM209 | 4 | 145-175 | 0.542 | 0.6121 | 0.000 | 0.552 | 1.000 |
| 111 | RM245 | 4 | 145-155 | 0.583 | 0.5772 | 0.000 | 0.518 | 1.000 |
| 112 | RM1089 | 4 | 210-260 | 0.417 | 0.6367 | 0.067 | 0.565 | 0.896 |
| 113 | RM228 | 4 | 110-170 | 0.625 | 0.5440 | 0.192 | 0.491 | 0.650 |
| 114 | RM401 | 3 | 250-300 | 0.754 | 0.3979 | 0.058 | 0.360 | 0.855 |
| 115 | RM11 | 3 | 140-160 | 0.463 | 0.5902 | 0.008 | 0.502 | 0.986 |
| 116 | RM3351 | 3 | 170-190 | 0.583 | 0.5174 | 0.000 | 0.420 | 1.000 |
| 117 | RM5749 | 3 | 130-160 | 0.588 | 0.5041 | 0.025 | 0.400 | 0.951 |
| 118 | RM335 | 2 | 100-110 | 0.721 | 0.4025 | 0.075 | 0.321 | 0.815 |
| 119 | RM144 | 3 | 200-210 | 0.588 | 0.5156 | 0.158 | 0.419 | 0.695 |
| 120 | RM300 | 3 | 125-145 | 0.867 | 0.2378 | 0.017 | 0.221 | 0.930 |
| 121 | RM1132 | 4 | 90-125 | 0.358 | 0.7245 | 0.033 | 0.674 | 0.954 |
| 122 | RM400 | 4 | 210-260 | 0.367 | 0.7172 | 0.467 | 0.665 | 0.353 |
| 123 | RM471 | 3 | 100-120 | 0.800 | 0.3378 | 0.000 | 0.309 | 1.000 |
| 124 | RM243 | 3 | 120-140 | 0.575 | 0.5538 | 0.017 | 0.475 | 0.970 |
| 125 | RM467 | 3 | 200-210 | 0.558 | 0.5754 | 0.000 | 0.502 | 1.000 |
| 126 | RM564 | 4 | 250-300 | 0.450 | 0.5994 | 0.100 | 0.515 | 0.834 |
| 127 | RM8007 | 3 | 130-150 | 0.767 | 0.3849 | 0.000 | 0.352 | 1.000 |
| 128 | RM441 | 4 | 160-200 | 0.475 | 0.6271 | 0.567 | 0.557 | 0.100 |
| 129 | RM518 | 3 | 150-170 | 0.542 | 0.5374 | 0.000 | 0.437 | 1.000 |
| 130 | RM253 | 4 | 130-170 | 0.554 | 0.5942 | 0.083 | 0.530 | 0.861 |
| 131 | RM274 | 3 | 75-80 | 0.667 | 0.4765 | 0.000 | 0.406 | 1.000 |
| 132 | RM242 | 4 | 200-240 | 0.575 | 0.5907 | 0.017 | 0.536 | 0.972 |
| 133 | RM3231 | 4 | 170-550 | 0.346 | 0.7033 | 0.650 | 0.645 | 0.080 |
| 134 | RM5687 | 4 | 160-500 | 0.417 | 0.6874 | 0.650 | 0.630 | 0.059 |
| 135 | RM5626 | 3 | 165-180 | 0.583 | 0.5117 | 0.733 | 0.411 | -0.430 |
| 136 | RM452 | 3 | 240-250 | 0.475 | 0.6182 | 0.000 | 0.541 | 1.000 |
|  | Mean | 4 |  | 0.561 | 0.5545 | 0.114 | 0.496 | 0.795 |
