# Supplementary material for: Unraveling the genomic regions controlling the seed vigour index, root growth parameters and germination per cent in rice
Source: PLoS One. 2022 Jul 26;17(7):e0267303. doi: 10.1371/journal.pone.0267303 (PMC9321372; doi:10.1371/journal.pone.0267303)
Supplement: S4 Table — (DOCX) [file pone.0267303.s006.docx]

**Supplementary Table 4**. Significant marker-trait associations detected for seed vigour index, root growth parameters and germination per cent by GLM approach at p<0.01.

| Trait | Marker | Position | marker_F | marker_p | marker_Rsq |
| --- | --- | --- | --- | --- | --- |
| GP | RM3701 | 48 | 8.32633 | 0.00466 | 0.05001 |
| GP | RM14723 | 87 | 8.73022 | 0.00379 | 0.05226 |
| GP | RM225 | 93 | 10.41636 | 0.00162 | 0.06153 |
| GP | RM20A | 128 | 7.10002 | 0.00881 | 0.04307 |
| GP | RM7179 | 159 | 9.05346 | 0.00322 | 0.05406 |
| GP | RM502 | 178 | 15.17228 | 1.65E-04 | 0.08637 |
| SVI-1 | RM3701 | 46 | 14.73858 | 2.02E-04 | 0.06101 |
| SVI-1 | RM103 | 90 | 7.08407 | 0.00888 | 0.03115 |
| SVI-1 | RM5436 | 137 | 9.5638 | 0.00249 | 0.04122 |
| SVI-1 | RM556 | 172 | 7.05373 | 0.00902 | 0.03102 |
| SVI-1 | RM502 | 177 | 8.4195 | 0.00444 | 0.03662 |
| SVI-1 | RM502 | 178 | 15.66579 | 1.31E-04 | 0.06439 |
| SVI-1 | RM336 | 226 | 8.02746 | 0.00544 | 0.03503 |
| SVI-1 | RM13600 | 260 | 7.6846 | 0.00649 | 0.03362 |
| SVI-1 | RM274 | 357 | 7.3337 | 0.00779 | 0.03218 |
| SVI-2 | RM13335 | 8 | 10.66622 | 0.00143 | 0.05185 |
| SVI-2 | RM85 | 17 | 9.15232 | 0.00306 | 0.04503 |
| SVI-2 | RM328 | 26 | 8.80412 | 0.00365 | 0.04344 |
| SVI-2 | RM337 | 27 | 9.30595 | 0.00283 | 0.04573 |
| SVI-2 | RM506 | 43 | 7.98991 | 0.00554 | 0.03968 |
| SVI-2 | RM440 | 65 | 11.2514 | 0.00108 | 0.05444 |
| SVI-2 | RM1347 | 82 | 8.06658 | 0.00533 | 0.04003 |
| SVI-2 | RM103 | 90 | 12.04771 | 7.29E-04 | 0.05793 |
| SVI-2 | RM405 | 109 | 11.75245 | 8.42E-04 | 0.05664 |
| SVI-2 | RM148 | 151 | 10.61885 | 0.00147 | 0.05164 |
| SVI-2 | RM7179 | 159 | 9.76818 | 0.00224 | 0.04782 |
| SVI-2 | RM502 | 177 | 8.70157 | 0.00385 | 0.04296 |
| SVI-2 | RM502 | 178 | 14.43461 | 2.33E-04 | 0.06814 |
| SVI-2 | RM3 | 190 | 10.93544 | 0.00126 | 0.05304 |
| SVI-2 | RM168 | 199 | 7.0591 | 0.009 | 0.03532 |
| SVI-2 | RM4112 | 209 | 9.55635 | 0.0025 | 0.04686 |
| SVI-2 | RM441 | 348 | 14.81491 | 1.95E-04 | 0.06973 |
| RRG | RM582 | 5 | 8.90403 | 0.00347 | 0.0424 |
| RRG | RM222 | 18 | 16.71582 | 8.04E-05 | 0.07491 |
| RRG | RM337 | 27 | 14.65199 | 2.10E-04 | 0.06669 |
| RRG | RM22034 | 56 | 8.73532 | 0.00378 | 0.04165 |
| RRG | RM223 | 60 | 23.21457 | 4.42E-06 | 0.09917 |
| RRG | RM405 | 109 | 10.5029 | 0.00156 | 0.04938 |
| RRG | RM7179 | 159 | 12.08155 | 7.17E-04 | 0.0561 |
| RRG | RM3375 | 200 | 7.31372 | 0.00787 | 0.03527 |
| RRG | RM494 | 221 | 11.71997 | 8.55E-04 | 0.05457 |
| RRG | RM494 | 222 | 16.10333 | 1.07E-04 | 0.0725 |
| RRG | RM5638 | 284 | 8.52708 | 0.00421 | 0.04072 |
| RRG | RM16686 | 297 | 11.58296 | 9.14E-04 | 0.05399 |
| RRG | RM243 | 339 | 7.28598 | 0.00799 | 0.03515 |
| RRG | RM243 | 340 | 10.66517 | 0.00144 | 0.05007 |
| RRG | RM441 | 349 | 11.23543 | 0.00108 | 0.05252 |
| RPE | RM24161 | 57 | 8.04519 | 0.00539 | 0.05889 |
| RPE | RM25181 | 140 | 16.20441 | 1.02E-04 | 0.1113 |
| RPE | RM403 | 184 | 12.45638 | 5.98E-04 | 0.08805 |
| RPE | RM309 | 185 | 10.5813 | 0.0015 | 0.07591 |
| RPE | RM309 | 186 | 7.46646 | 0.00727 | 0.05491 |
| RPE | RM1341 | 208 | 8.35735 | 0.00459 | 0.06103 |
| RPE | RM233 | 254 | 8.851 | 0.00356 | 0.06437 |
| RPE | RM13600 | 260 | 9.95029 | 0.00205 | 0.07174 |
| RPE | RM6091 | 302 | 7.2951 | 0.00795 | 0.05373 |
| RPE | RM518 | 351 | 7.8548 | 0.00594 | 0.05759 |
| RPE | RM518 | 352 | 7.91619 | 0.00576 | 0.05801 |
| RSR | RM3701 | 46 | 9.14305 | 0.00307 | 0.07096 |
| RSR | RM216 | 70 | 7.25683 | 0.00811 | 0.05718 |
| RSR | RM405 | 109 | 14.45053 | 2.31E-04 | 0.10758 |
| RSR | RM6641 | 187 | 13.09154 | 4.41E-04 | 0.09849 |
| RSR | RM168 | 199 | 10.34346 | 0.00168 | 0.07951 |
| RSR | RM6374 | 247 | 9.08219 | 0.00317 | 0.07052 |
| RSR | RM144 | 328 | 7.064 | 0.00897 | 0.05575 |
