# Supplementary material for: Unraveling the genomic regions controlling the seed vigour index, root growth parameters and germination per cent in rice
Source: PLoS One. 2022 Jul 26;17(7):e0267303. doi: 10.1371/journal.pone.0267303 (PMC9321372; doi:10.1371/journal.pone.0267303)
Supplement: S5 Table — (DOCX) [file pone.0267303.s007.docx]

**Supplementary Table 5**. Significant marker-trait associations detected for seed vigour index, root parameters and germination per cent parameters by MLM approach at p<0.01

| Trait | Marker | Pos | F | p | Marker_R2 |
| --- | --- | --- | --- | --- | --- |
| GP | RM506 | 43 | 7.78363 | 0.00617 | 0.06181 |
| GP | RM225 | 93 | 8.80688 | 0.00365 | 0.06994 |
| GP | RM7179 | 159 | 9.82798 | 0.00218 | 0.07805 |
| GP | RM502 | 178 | 7.06356 | 0.00898 | 0.0561 |
| GP | RM3 | 190 | 8.0464 | 0.00538 | 0.0639 |
| SVI-1 | RM3701 | 46 | 8.02083 | 0.00545 | 0.05653 |
| SVI-1 | RM502 | 178 | 7.21569 | 0.00829 | 0.05085 |
| SVI-1 | RM13600 | 258 | 7.15409 | 0.00856 | 0.05042 |
| SVI-1 | RM253 | 353 | 10.0857 | 0.00191 | 0.07108 |
| SVI-2 | RM13335 | 8 | 7.02387 | 0.00917 | 0.05385 |
| SVI-2 | RM337 | 27 | 9.71925 | 0.0023 | 0.07452 |
| SVI-2 | RM14723 | 87 | 7.99728 | 0.00552 | 0.06132 |
| SVI-2 | RM103 | 90 | 11.38112 | 0.00101 | 0.08726 |
| SVI-2 | RM7364 | 115 | 7.45172 | 0.00733 | 0.05714 |
| SVI-2 | RM3 | 190 | 11.14453 | 0.00113 | 0.08545 |
| SVI-2 | RM5638 | 282 | 9.30066 | 0.00284 | 0.07131 |
| SVI-2 | RM8007 | 346 | 7.63719 | 0.00665 | 0.05856 |
| SVI-2 | RM441 | 348 | 7.23687 | 0.0082 | 0.05549 |
| RRG | RM222 | 18 | 9.10484 | 0.00313 | 0.06625 |
| RRG | RM337 | 27 | 8.37216 | 0.00455 | 0.06092 |
| RRG | RM223 | 60 | 19.27957 | 2.51E-05 | 0.14029 |
| RRG | RM405 | 109 | 7.62752 | 0.00669 | 0.0555 |
| RRG | RM7179 | 159 | 7.62911 | 0.00668 | 0.05551 |
| RRG | RM494 | 221 | 10.30089 | 0.00172 | 0.07495 |
| RRG | RM494 | 222 | 11.23597 | 0.00108 | 0.08176 |
| RRG | RM16686 | 297 | 10.85419 | 0.00131 | 0.07898 |
| RRG | RM243 | 340 | 8.17956 | 0.00503 | 0.05952 |
| RPE | RM14978 | 51 | 8.00961 | 0.00549 | 0.06743 |
| RPE | RM494 | 221 | 7.00362 | 0.00926 | 0.05896 |
| RSR | RM3701 | 48 | 8.27567 | 0.00478 | 0.06994 |
| RSR | RM405 | 109 | 11.65994 | 8.81E-04 | 0.09854 |
| RSR | RM6641 | 187 | 8.78196 | 0.00369 | 0.07421 |
| RSR | RM168 | 199 | 7.7835 | 0.00617 | 0.06578 |
| RSR | RM3231 | 363 | 9.70314 | 0.00232 | 0.082 |
